# Working towards recalcitrance mechanisms: increased xylan and homogalacturonan production by overexpression of *GAlactUronosylTransferase12* (*GAUT12*) causes increased recalcitrance and decreased growth in *Populus*

**DOI:** 10.1186/s13068-017-1002-y

**Published:** 2018-01-17

**Authors:** Ajaya K. Biswal, Melani A. Atmodjo, Sivakumar Pattathil, Robert A. Amos, Xiaohan Yang, Kim Winkeler, Cassandra Collins, Sushree S. Mohanty, David Ryno, Li Tan, Ivana Gelineo-Albersheim, Kimberly Hunt, Robert W. Sykes, Geoffrey B. Turner, Angela Ziebell, Mark F. Davis, Stephen R. Decker, Michael G. Hahn, Debra Mohnen

**Affiliations:** 1 Department of Biochemistry and Molecular Biology, University of Georgia, Athens, GA 30602 USA; 2 Department of Plant Biology, University of Georgia, Athens, GA 30602 USA; 3 Complex Carbohydrate Research Center, University of Georgia, 315 Riverbend Rd., Athens, GA 30602-4712 USA; 4 DOE-BioEnergy Science Center (BESC), Oak Ridge National Laboratory, Oak Ridge, TN 37831 USA; 5 Bioscience Division, Oak Ridge National Laboratory, Oak Ridge, TN 37831 USA; 6 ArborGen, Inc., 2011 Broadbank Ct., Ridgeville, SC 29472 USA; 7 National Renewable Energy Laboratory, Golden, CO 80401-3305 USA; 8 Present Address: Mascoma LLC (Lallemand Inc.), 67 Etna Rd., Lebanon, NH 03766 USA; 9 Present Address: South Georgia State College, 100 West College Park Dr., Douglas, GA 31533 USA; 10 Present Address: Nuclear Materials Science, Los Alamos National Laboratory, P.O. Box 1663, Los Alamos, NM 87545-1663 USA; 11 Present Address: Nu Mark LLC, 6601 W. Broad St., Richmond, VA 23230 USA

**Keywords:** Plant cell wall, Biofuel, Biomass, Pectin, Xylan, Yield

## Abstract

**Background:**

The development of fast-growing hardwood trees as a source of lignocellulosic biomass for biofuel and biomaterial production requires a thorough understanding of the plant cell wall structure and function that underlie the inherent recalcitrance properties of woody biomass. Downregulation of *GAUT12.1* in *Populus deltoides* was recently reported to result in improved biomass saccharification, plant growth, and biomass yield. To further understand *GAUT12.1* function in biomass recalcitrance and plant growth, here we report the effects of *P. trichocarpa GAUT12.1* overexpression in *P. deltoides*.

**Results:**

Increasing *GAUT12.1* transcript expression by 7–49% in *P. deltoides PtGAUT12.1*-overexpression (OE) lines resulted in a nearly complete opposite biomass saccharification and plant growth phenotype to that observed previously in *PdGAUT12.1*-knockdown (KD) lines. This included significantly reduced glucose, xylose, and total sugar release (12–13%), plant height (6–54%), stem diameter (8–40%), and overall total aerial biomass yield (48–61%) in 3-month-old, greenhouse-grown *PtGAUT12.1*-OE lines compared to controls. Total lignin content was unaffected by the gene overexpression. Importantly, selected *PtGAUT12.1*-OE lines retained the recalcitrance and growth phenotypes upon growth for 9 months in the greenhouse and 2.8 years in the field. *PtGAUT12.1*-OE plants had significantly smaller leaves with lower relative water content, and significantly reduced stem wood xylem cell numbers and size. At the cell wall level, xylose and galacturonic acid contents increased markedly in total cell walls as well as in soluble and insoluble cell wall extracts, consistent with increased amounts of xylan and homogalacturonan in the *PtGAUT12.1*-OE lines. This led to increased cell wall recalcitrance, as manifested by the 9–15% reduced amounts of recovered extractable wall materials and 8–15% greater amounts of final insoluble pellet in the *PtGAUT12.1*-OE lines compared to controls.

**Conclusions:**

The combined phenotype and chemotype data from *P. deltoides PtGAUT12.1*-OE and *PdGAUT12.1*-KD transgenics clearly establish *GAUT12.1* as a recalcitrance- and growth-associated gene in poplar. Overall, the data support the hypothesis that GAUT12.1 synthesizes either an HG-containing primer for xylan synthesis or an HG glycan required for proper xylan deposition, anchoring, and/or architecture in the wall, and the possibility of HG and xylan glycans being connected to each other by a base-sensitive covalent linkage.

**Electronic supplementary material:**

The online version of this article (10.1186/s13068-017-1002-y) contains supplementary material, which is available to authorized users.

## Background

Development of strategies to deconstruct lignocellulosic biomass for biofuels and biomaterials production is essential to advance a sustainable economy and to mitigate greenhouse gas emission-related climate change [1, 2]. Trees accumulate a major portion of terrestrial biomass as secondary cell walls that account for a substantial amount of global carbon sequestration [3, 4]. Hardwood biomass is a complex polymeric matrix of cellulose, hemicellulose (primarily xylan), and lignin along with significant amounts of pectin [5, 6]. The structural diversity of the different wall polymers, and the inter- and intramolecular interactions via covalent and non-covalent linkages among them, influence the mechanical and chemical properties of the biomass that are important for tree survival and for quality (e.g., fiber length, fiber strength) of wood-derived materials such as timber, paper, cellulose, lignin, and others [7, 8]. Understanding the structural complexity, interaction, and functionality of the cell wall polymers is therefore essential for unraveling the molecular basis of biomass recalcitrance and plant growth, and to generate by biotechnological manipulation improved biomass with reduced recalcitrance and high yield.

**Fig. 1:**
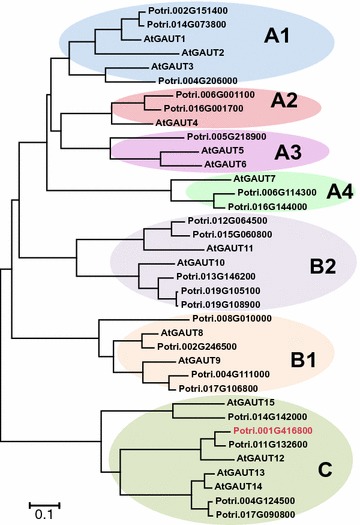
A phylogenetic tree of the GAUT protein family of *Arabidopsis thaliana* (TAIR10) and *Populus trichocarpa* (Phytozome 11.0; *Populus trichocarpa* v3.0), showing the relationship between amino acid sequences. Potri.001G416800 (in red font) is named in this paper as *Pt*GAUT12.1. The tree was constructed by the Neighbor-Joining method using MEGA6 [48]

In prior research, we identified a recalcitrance-associated gene, *GAlactUronosylTransferase (GAUT)12* whose modified expression in poplar led to both reduced biomass recalcitrance and increased plant growth [5]. *GAUT12* is a putative galacturonosyltransferase (GalAT) belonging to the *GAUT* gene family (Fig. 1) within the glycosyltransferase (GT) 8 family [9, 10]. *GAUT12* was first identified as a gene involved in *Arabidopsis thaliana* (*At*) secondary wall formation [11, 12]. It is highly expressed in stems and roots, particularly in cells undergoing secondary wall thickening including interfascicular fibers and primary and secondary xylem [11, 13]. Arabidopsis *irregular xylem8* (*irx8*) mutants, defective in the *GAUT12* gene [11, 12] are severely dwarfed, semi-sterile due to indehiscent anthers and have a collapsed xylem vessel phenotype [13–15]. Compared to wild type (WT), Arabidopsis *irx8* mutant cell walls have a greater than 50% reduction in glucuronoxylan (GX) and an almost complete absence of the β-d-Xyl*p*-(1,3)-α-l-Rha*p*-(1,2)-α-d-Gal*p*A-(1,4)-d-Xyl*p* xylan reducing end tetrasaccharide sequence, indicating a critical role of *AtGAUT12* in xylan biosynthesis [11, 13, 14, 16]. However, significantly decreased amounts of pectin were also observed in pectin-enriched wall fractions from *irx8* mutants compared to WT [14, 16], leading to the hypothesis that *AtGAUT12* is involved in either the insertion of GalA into the xylan reducing end sequence, or in the synthesis of a subfraction of homogalacturonan (HG) [14] required for xylan synthesis. Arabidopsis stem lignin content was also reduced in the *irx8* mutants, and immunohistochemistry of stem sections using multiple anti-xylan monoclonal antibodies revealed different xylan localization patterns between the *irx8* mutants and WT [15, 17], suggesting a role for the GAUT12-synthesized cell wall polymer in wall architecture. Based on the data from Arabidopsis, the results suggest that *GAUT12* functions in the synthesis of a structure required for xylan and lignin deposition during secondary cell wall formation in Arabidopsis, and that the structure either contains, or is dependent upon, an HG-containing glycan.

Since several other members of the *GAUT* gene family have been shown to have homogalacturonan:galacturonosyltransferase (HG:GalAT) activity [10, 18], GAUT12 was hypothesized to also have GalAT activity. GAUT12 is predicted to be a type II membrane protein and has been shown to localize to the Golgi in both Arabidopsis and poplar [13, 19]. In a study designed to identify the enzyme function of GAUT12, it was shown that the Arabidopsis *irx8* mutant did not have reduced xylan xylosyltransferase (XylT) or xylan glucuronosyltransferase (GlcAT) activities [16, 20], thereby providing no support for a function of GAUT12 directly in xylan synthesis. On the contrary, Hao et al. [15] identified ~ 45% reduced HG:GalAT activity in microsomes from *irx8/gaut12* stems compared to WT, suggesting a possible function of GAUT12 in HG synthesis. However, no HG:GalAT activity was detected from GAUT12-immunoabsorbed from WT solubilized microsomes [15] when a typical HG:GalAT enzyme assay was used [10, 21]. While it is possible that the standard HG:GalAT reaction conditions (e.g., exogenous acceptor used) and/or the amount or condition of the immunopurified Arabidopsis GAUT12 was insufficient to detect HG:GalAT activity in vitro from the immunopurified Arabidopsis GAUT12, the role of GAUT12 in xylan biosynthesis remains to be determined.

Poplar has two homologs of *AtGAUT12*, designated *GAUT12.1* (*Potri.001G416800*, Genemodel V3.0; Phytozome 12.0) and *GAUT12.2* (*Potri.011G132600*, Genemodel V3.0; Phytozome 12.0) that are 91 and 90% identical to each other in their amino acid and nucleotide sequences, respectively. Both genes are expressed in poplar stem primary and differentiating xylem, secondary xylem, and phloem fibers, with *GAUT12.1* expression being seven times greater than *GAUT12.2* [19, 22]. Simultaneous downregulation of both genes in *Populus trichocarpa* [22] and *Populus alba x tremula* [23] significantly reduced the transcript level of both genes and resulted in 20–40% decreased stem xylan content compared to controls, consistent with a function of *GAUT12* in xylan biosynthesis. The xylan reducing end tetrasaccharide sequence was also reduced in the *GAUT12* knockdown (KD) transgenics compared to WT in the *P. alba x tremula* study [23]. However, in contrast to Arabidopsis-dwarfed *irx8* mutants, the transgenic double *GAUT12.1/GAUT12.2*-knocked down poplar plants did not show reduced growth or collapsed xylem phenotypes, although they had thinner cell walls and, in one study, slightly deformed vessel cells [22, 23]. Furthermore, lignin content was increased in the *P. trichocarpa GAUT12*-KD wood biomass [22], but was reduced in the *P. alba x tremula GAUT12*-KD samples [23].

Recently, we reported the specific downregulation of only the *GAUT12.1* gene in *P. deltoides* [5] and described the consequences of this genetic manipulation on plant/wood growth and development and biomass saccharification efficiency. *PdGAUT12.1* was selected due to its greater transcript abundance than *PdGAUT12.2.* As expected, the cell walls of *PdGAUT12.1*-KD plants were significantly reduced in xylose (Xyl) and galacturonic acid (GalA) content, in comparison to control plants. These results indicated that *PdGAUT12.1* is involved in xylan and pectin formation in *Populus*, in a similar fashion to *AtGAUT12* in Arabidopsis. Wood from the *PdGAUT12.1*-KD lines also had reduced recalcitrance compared to control lines [5]. In agreement with this finding, a recent study of segregating *Eucalyptus* hybrid tree populations using network-based data integration methodology revealed the association of *GAUT12* with sugar release traits [24]. Contrary to the *P. trichocarpa* and *P. alba x tremula* double homolog knockdown transgenics described above, however, *PdGAUT12.1*-KD lines showed no change in the total lignin content [5]. Most importantly, *PdGAUT12.1*-KD plants had larger cell size, growth, and biomass yield compared to the WT [5], which is in contrast to the negative or neutral growth phenotypes of the Arabidopsis *irx8* knockout mutants and the poplar double homolog knockdown transgenics [22, 23]. Overall, the results support the hypothesis that GAUT12 is required for the synthesis of a native xylan-containing polymer, but also suggest that there is a fine balance between the amount and/or structure of that polymer, wall structural properties and plant growth.

Despite the above-described extensive research on the *gaut12/irx8* mutants and the *GAUT12* gene and transgenics to date, the exact biochemical and biological function of GAUT12 remains unsolved. It is also unclear why the lack of *GAUT12* expression inhibits growth so severely in Arabidopsis *irx8* knockout mutants [11–14], but the simultaneous reduced expression of *GAUT12.1* and *GAUT12.2* in poplar does not negatively impact growth [22, 23] and the reduced expression of only *GAUT12.1* increases growth in *P. deltoides* [5].

The goal of the research reported here was to understand the biological function of GAUT12 in poplar wood, and the mechanism for how modified *GAUT12* expression affects biomass recalcitrance and growth. To this end, we overexpressed *P. trichocarpa GAUT12.1* (*PtGAUT12.1*) in *P. deltoides* and characterized the transgenic plants for recalcitrance and growth phenotypes. We hypothesized that in the resulting *PtGAUT12.1* overexpression (OE) lines, we would obtain one of the two results: (1) GAUT12 enzyme function would require coordinated expression of multiple genes, in which case overexpression of GAUT12 alone would not increase the expression of the synthesized polymers and therefore no recalcitrance/growth phenotype would be manifested, or (2) GAUT12 overexpression would increase the amount of GAUT12-synthesized polymers, resulting in associated phenotypes/chemotypes of the plant and cell walls. The latter possibility would enable the analyses of such modified cell walls with the goal of obtaining further insight into the biological and cell wall/enzyme function of GAUT12. Here we report that overexpression of *PtGAUT12.1* yields *PtGAUT12.1*-OE lines with the opposite growth, recalcitrance, and cell wall phenotypes as those observed in the *P. deltoides GAUT12.1*-knockdown (*PdGAUT12.1*-KD) lines [5]. Analysis of the *PtGAUT12.1*-OE biomass and phenotypes, and comparison of these results with the chemotypes/phenotypes of the previously reported *PdGAUT12.1*-KD data provide a comprehensive dataset that strongly supports the hypothesis that GAUT12 functions in the synthesis of a xylan- and homogalacturonan-containing polymer that has roles in cell wall integrity, biomass recalcitrance, and plant growth in woody feedstock.

## Results

### *Populus GAUT12.1* is expressed in the shoot apex, young developing leaves, and internodes, as well as in secondary wall-rich stem and root tissues, indicating a broader role for GAUT12 than only in secondary walls

**Fig. 2:**
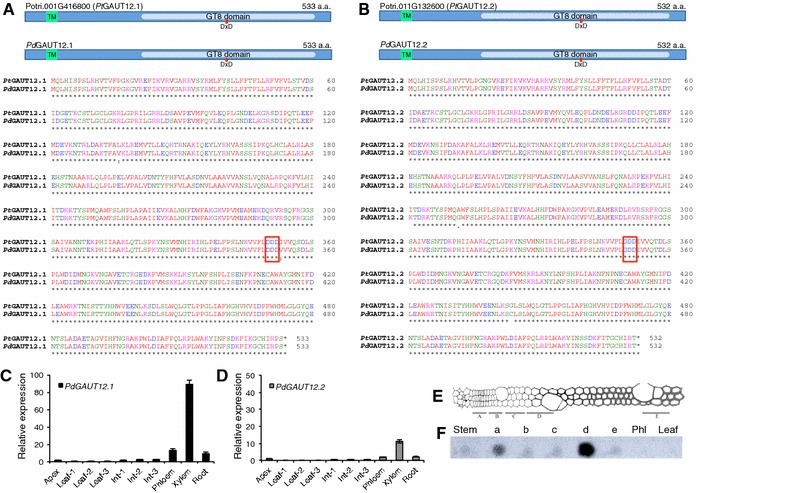
Protein sequence alignment and transcript expression of *PdGAUT12*. **A**, **B** Schematic and alignment of *P. trichocarpa* and *P. deltoides* GAUT12.1 and GAUT12.2 proteins, respectively. *P. deltoides GAUT12.1* and *GAUT12.2* were previously cloned [5] (Genbank accession numbers MG656447 and MG656448, respectively). The N-terminal cytoplasmic domain and the transmembrane domain (TM) were predicted using TMHMM v. 2.0 [49]. The glycosyltransferase family 8 (GT8) domain (PF01501) and the predicted catalytic domain DxD motif are noted. **C**, **D** Relative expressions of *PdGAUT12.1* and *PdGAUT12.2*, respectively, in different tissues of *P. deltoides* as determined by quantitative RT-PCR. Data represent means ± standard error of three biological replicates and two technical replicates, *n* = 6. **E** Schematic representation of different stages of wood development in *Populus* wood (reprinted with permission from [50] [Copyright (2001) National Academy of Sciences, USA]. **F** Dot blot measurement of *PdGAUT12.1* transcript abundance in different tissues and wood development zones (a–e) of *P. deltoides*. A radiolabeled probe corresponding to *PdGAUT12.1* cDNA was hybridized onto a membrane blotted using equal amounts of total RNA from each tissue. The results are representative of 3 independent dot blots. a: Vascular cambium; b: expansion zone; c: transition zone; d: secondary wall formation zone; e: cell death zone; Phl: phloem

Previous studies of *GAUT12* primarily emphasized its function in secondary cell walls, focusing mostly on stem tissues (in Arabidopsis) [13–16] and woody biomass (in poplar) [5, 22, 23]. High *GAUT12* expression was found in xylem while low levels of expression were found in tissues such as anther, pollen, leaf vascular tissue, and hypocotyls [11, 14, 15]. Low expression of *Pt* and *PdGAUT12.1* was also reported in poplar leaves [5, 22]. To confirm the broad expression of *GAUT12* and to dissect its expression in young tissues, we analyzed *PdGAUT12.1* and *PdGAUT12.2* expression in developing organs of *P. deltoides* by quantitative RT-PCR. Xylem tissues were included as a control. As expected, xylem tissues had the highest levels of expression of both the *PdGAUT12.1* and *PdGAUT12.2* genes (Fig. 2C, D). However, *PdGAUT12.1* expression was also evident, albeit at much lower levels, in the phloem and root tissues, and expression was detected at lower but clearly measurable levels in the apex, the first three leaves, and the first three internodes from the top of the plant (Fig. 2C). Similar but lower expression was observed for *PdGAUT12.2*, although no expression was detectable in leaves 1, 2, and 3 (Fig. 2D).

We also studied *PdGAUT12.1* expression in the different developmental zones of poplar wood by RNA blot analysis using a 3′-UTR nucleotide gene probe (Fig. 2E, F). *PdGAUT12.1* was expressed very strongly in the secondary wall formation zone, and also in a lower but substantial amount in the vascular cambium. Much lower expression was detected in the expansion, transition, and cell death zones as well as in whole stem tissue. No *PdGAUT12.1* expression was detectable in phloem and leaf tissues by this RNA blot method.

### Overexpression of *PtGAUT12.1* in *Populus deltoides*

At the conception of this work, the sequenced *P. trichocarpa* genome was available and used as the poplar reference genome within the BioEnergy Science Center (BESC). The genome information of *P. deltoides*, the poplar species used as genetic background for transgenesis in BESC, was at that time unavailable. Therefore, we decided to clone and overexpress *P. trichocarpa GAUT12.1* in *P. deltoides*. Both the *P. deltoides GAUT12.1* and *GAUT12.2* were later cloned for the purpose of complementation of Arabidopsis *irx8* mutants, the results of which have been reported previously [5]. Comparison of *P. trichocarpa GAUT12.1* and *GAUT12.2* with their cloned *P. deltoides* counterparts (Fig. 2A, B) showed that both sets of orthologs share 99% sequence identity at both the protein and nucleotide levels. Recently, a pre-released version of *P. deltoides* genome has become available through Phytozome 12 (https://phytozome.jgi.doe.gov). It is noteworthy, however, that while Phytozome *Podel.11G130300.1* coding sequence matches 99% to the cloned *PdGAUT12.2*, *Podel.01G434500.1* coding sequence seems to be incomplete, and thus matches the cloned *PdGAUT12.1* by only 94%. Based on the relatively high sequence similarity between the *PtGAUT12.1* and *PdGAUT12.1*, we expected that overexpression of the former in *P. deltoides* would produce similar phenotypic effects as would overexpression of the latter.

**Fig. 3:**
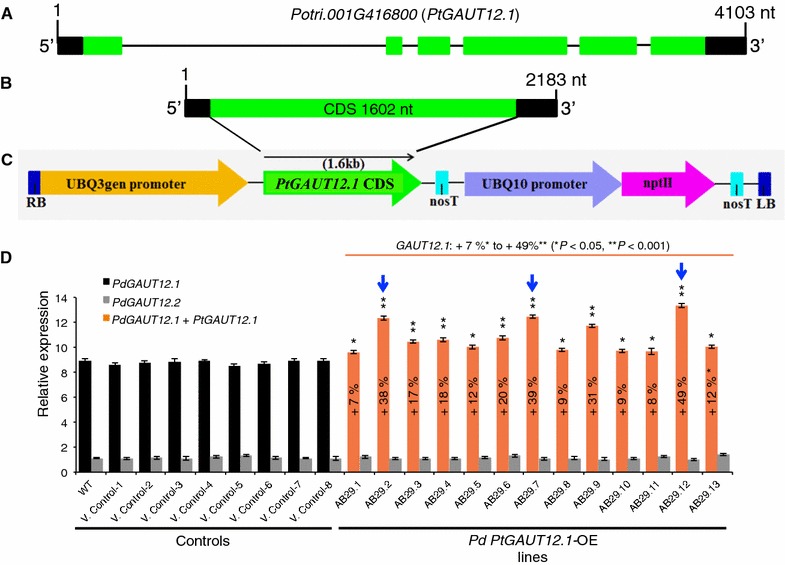
*PtGAUT12.1* gene model, overexpression vector map, and *GAUT12.1* transcript abundance in *P. deltoides PtGAUT12*-OE lines. **A** *PtGAUT12.1* (Potri.001G416800) gene model from Phytozome 11.0 *Populus trichocarpa* v3.0. nt, nucleotides. **B** Structure of *PtGAUT12.1* (Potri.001G416800) mRNA. CDS, coding sequence. **C** A schematic of the *PtGAUT12.1* (Potri.001G416800) overexpression construct used to generate *P. deltoides PtGAUT12.1* overexpression lines. **D** Relative *GAUT12.1* and *GAUT12.2* transcript abundance as determined by quantitative RT-PCR analysis of 3-month-old *P. deltoides* wild-type (WT) and *PtGAUT12.1*-OE lines. The *18S rRNA* was used as the reference gene and the transcript expression of *PdGAUT12.2* in AB29.12 was set to 1. Error bars represent SE, *n* = 6, *P < 0.05, **P < 0.001. Blue arrows indicate the three *P. deltoides PtGAUT12.1*-OE lines (AB29.2, AB29.7, and AB29.12) selected for further analyses

An overexpression construct containing *P. trichocarpa GAUT12.1* coding sequence (1602 bp) driven by *A. thaliana Ubiquitin3* promoter (Fig. 3A–C) was introduced into the *P. deltoides* clone WV94 background. Thirteen *PtGAUT12.1*-overexpression (OE) transgenic lines (AB29.1 through AB29.13) were generated with the presence of the transgene confirmed by PCR in each of the lines (data not shown). For this study, 10–15 clones of each of the thirteen *PtGAUT12.1*-OE lines were analyzed along with 25 non-transformed wild-type (WT) plants and 10–15 clones of eight independent vector control lines (V. Control-1 through 8).

*GAUT12.1* and *GAUT12.2* transcript expression in the *PtGAUT12.1*-OE lines compared to controls was investigated using quantitative RT-PCR with primer pairs matching both endogenous *PdGAUT12.1* and the transgene *PtGAUT12.1*. Total *GAUT12.1* transcript expression was increased by 7–49% in all thirteen OE lines compared to the WT and vector controls (Fig. 3D). Based on the extent of transcript overexpression, the *PtGAUT12.1*-OE lines were divided into three groups: lines AB29.1, AB29.8, AB29.10, and AB29.11 had 7–9% increased transcript levels; lines AB29.3, AB29.4, AB29.5, AB29.6, and AB29.13 lines had 12–20% increased transcript levels; and AB29.2, AB29.7, AB29.9, and AB29.12 lines had 31–49% increased *GAUT12.1* transcript levels compared to the controls. As expected, *PdGAUT12.2* transcript expression in the OE lines was not affected by *PtGAUT12.1* overexpression and remained comparable to the controls (Fig. 3D).

### *PtGAUT12.1* overexpression inhibits saccharification but does not affect total lignin content

**Fig. 4:**
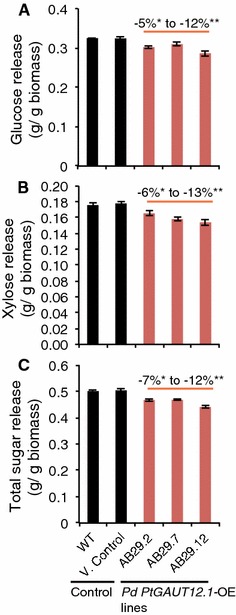
Saccharification yield of *P. deltoides PtGAUT12.1*-overexpression lines. **A** Glucose, **B** xylose, and **C** total sugar release from *P. deltoides* WT, vector control, and *PtGAUT12.1*-OE lines. Data are mean ± SE, *n* = 25 for WT, *n* = 120 for vector control (eight different insertion vector control lines, each with *n* = 15, see Additional file 1 for the full dataset), and *n* = 10–15 for the *PtGAUT12.1*-OE lines. Statistical analysis was by one-way analysis of variance (ANOVA) followed by Tukey’s multiple comparison test using Statistica 5.0

The effect of *PtGAUT12.1* overexpression on sugar release from *P. deltoides* wood was determined by subjecting wood biomass samples from 9-month-old control and *PtGAUT12.1*-OE trees to hot water pretreatment and enzymatic hydrolysis. Eight of the thirteen *PtGAUT12.1*-OE lines had 4–12% significantly decreased glucose release per gram dry biomass compared to WT and vector controls (Fig. 4A, Additional file 1A). Significant decreases were also observed for xylose release (5–13%; Fig. 4B, Additional file 1B) and total sugar release (4–12%; Fig. 4C, Additional file 1C) per gram dry biomass in six and five, respectively, of the thirteen transgenic lines compared to controls.

**Fig. 5:**
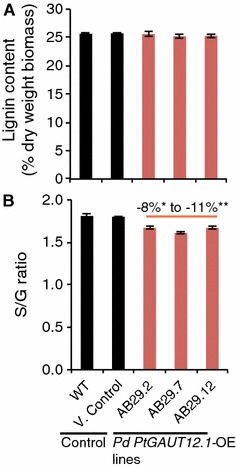
Total lignin content and S/G ratio of *P. deltoides PtGAUT12*-OE lines compared to controls. **A** Total lignin content and **B** S/G ratio of *P. deltoides* WT, vector control, and *PtGAUT12.1*-OE lines. Data are mean ± SE. *n* = 25 for WT, *n* = 120 for vector control (eight different insertion vector control lines, each with *n* = 15, see Additional file 2 for the full dataset), and *n* = 10–15 for *PtGAUT12.1*-OE lines, *P < 0.05, **P < 0.001. Statistical analysis was by one-way analysis of variance (ANOVA) followed by Tukey’s multiple comparison test using Statistica 5.0

We analyzed the amount of lignin in wood samples from all control and *PtGAUT12.1*-OE lines by pyrolysis molecular beam mass spectrometry. Total lignin content in the *PtGAUT12.1*-OE lines (25.2–26.3% in AB29.1–AB29.13) was similar to that in WT (25.7%) and vector controls (24.7–26.7% in V. Control-1 to -8) (Fig. 5A, Additional file 2). However, the lignin syringyl-to-guaiacyl (S/G) ratios were significantly decreased (8–11%) in five of the thirteen *PtGAUT12.1*-OE lines compared to those of WT and vector controls (Fig. 5B, Additional file 2).

### *PtGAUT12.1* overexpression decreases plant growth and biomass yield in *P. deltoides*

**Fig. 6:**
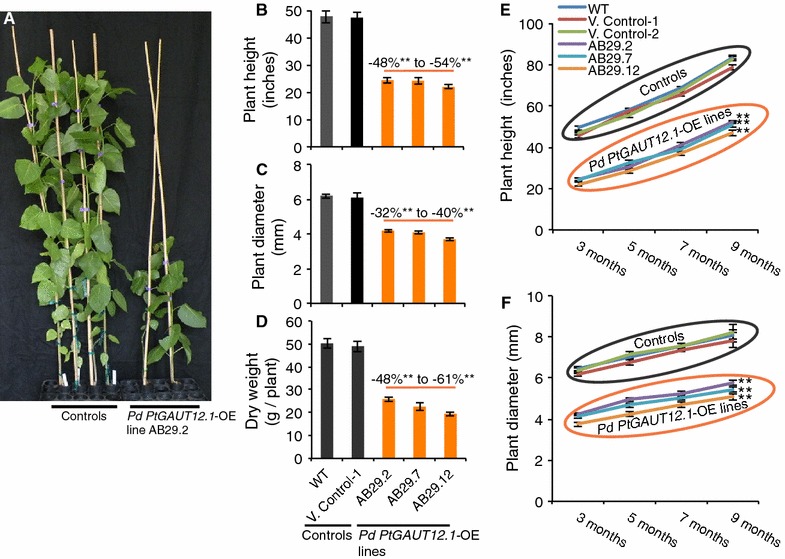
Growth phenotypes of *P. deltoides PtGAUT12.1*-OE lines. **A** Phenotypes of 3-month-old *P. deltoides* WT (left two plants of shown controls), vector control (right two plants of shown controls), and *PtGAUT12.1*-OE plants. **B** Height, **C** radial growth, and **D** dry aerial biomass weight of 3-month-old *PtGAUT12*-OE lines compared to WT and vector control. For height and diameter, *n* = 25 for WT, *n* = 120 for vector control lines (*n* = 10–15 for each of 8 control lines), and *n* = 10–15 for *PtGAUT12*-OE lines (complete dataset is provided in Additional file 3). For biomass weight, *n* = 6. **E** Height and **F** radial growth of greenhouse-grown *PtGAUT12*-OE and control plants measured over a 9-month growth period (*n* = 10). Error bars represent SE, *P < 0.05, **P < 0.001

Assessment of the effects of *PtGAUT12.1* overexpression on plant growth was first carried out on 3-month-old greenhouse-grown plants. Inhibition of vegetative plant growth was observed in the *PtGAUT12.1*-OE plants compared to the controls (Fig. 6A). Nine of the thirteen *PtGAUT12.1*-OE lines showed 6–54% significantly reduced plant height and 8–40% reduced stem radial diameter compared to WT and vector controls (Fig. 6B, C, Additional file 3). Both *PtGAUT12.1*-OE plant height and stem diameter were negatively correlated with the total *GAUT12.1* transcript expression (Additional file 4). This growth inhibition led to an overall 48–61% decrease in total aerial dry biomass of the greenhouse-grown, 3-month-old *PtGAUT12.1*-OE plants (Fig. 6D).

Three *PtGAUT12.1*-OE lines with the greatest increase in *GAUT12.1* transcript expression (AB29.2, AB29.7, and AB29.12; Fig. 3D) were selected for additional growth assessment in the greenhouse. Throughout the 9-month growing period, the three *PtGAUT12.1*-OE lines continued to exhibit reduced growth characteristics, including a 48–54% reduction in plant height and a 32–40% reduction in stem diameter compared to controls (Fig. 6E, F). Except for the field trial, further studies reported here were carried out on these three selected *PtGAUT12.1*-OE lines.

### Reduced growth and increased recalcitrance phenotypes are sustained in field-grown *PtGAUT12.1*-OE plants

**Fig. 7:**
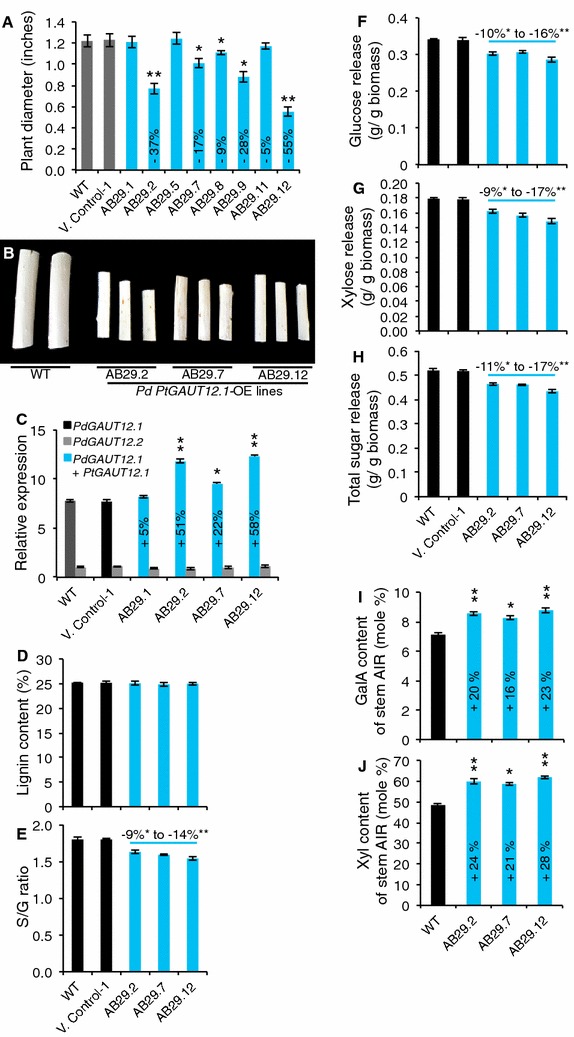
Plant diameter, transcript, lignin, saccharification, and cell wall composition of field-grown *P. deltoides* control and *PtGAUT12.1*-OE plants. **A** Plant diameter and **B** debarked stem radial diameter of field-grown *PtGAUT12.1*-OE plants compared to controls. **C** Relative abundance of *GAUT12.1* transcript determined by quantitative RT-PCR of RNA extracted from stems of 2.8-year-old field-grown trees and normalized to 18S. Expression of *PdGAUT12.2* in AB29.7 was set to 1. Each data point represents mean ± SD of two biological replicates and three technical replicates, *n* = 4. **D** Total lignin content and **E** S/G ratio of WT, vector control, and *PtGAUT12.1*-OE lines. **F** Glucose, **G** xylose, and **H** total sugar release from WT and transgenic lines. **I** Galacturonic acid (GalA) and **J** xylose (Xyl) contents of AIR from stem of 2.8-year-old field-grown WT and *PtGAUT12.1*-OE plants. *n* = 4. Error bars represent SE. *P < 0.05, **P < 0.001

A field trial was carried out to evaluate the stability of both the *PtGAUT12.1*-OE genetic modification and the associated phenotypes in the field environment. Seven *PtGAUT12.1*-OE lines (including AB29.2, AB29.7, and AB29.12) were grown alongside WT and vector control plants for 2.8 years in the field. At the end of the field trial, five of the seven *PtGAUT12.1*-OE lines had 9–55% smaller stem radial diameter compared to controls (Fig. 7A, B). The *PtGAUT12.1*-OE lines also had reduced height compared to the controls, based on visual observation. Quantitative RT-PCR analysis (Fig. 7C) again demonstrated the negative correlation between total *GAUT12.1* transcript abundance and plant growth.

Biomass samples harvested from the field-grown trees were also assessed for recalcitrance characteristics. Glucose, xylose, and total sugar release were all significantly reduced by 10–16, 9–17, and 11–17%, respectively, in the three *PtGAUT12.1*-OE lines compared to the controls (Fig. 7D–F). Similar to the greenhouse-grown trees, the field-grown *PtGAUT12.1*-OE lines were not affected in the total lignin content, but were reduced in lignin S/G ratios by 9–14% compared to the controls (Fig. 7G, H). Taken together, the results confirmed that the genetic manipulation and associated phenotypes were stably maintained in field-grown *PtGAUT12.1*-OE trees.

### *PtGAUT12.1* overexpression reduces leaf area and relative water content

The growth reduction in the *PtGAUT12.1*-OE lines was also manifested in smaller leaf size (Additional file 5A). To better evaluate this phenotype, we measured every third successive leaf from the apex down to leaf 25 in both *PtGAUT12.1*-OE and control lines. Both leaf length and width were significantly reduced in the *PtGAUT12.1*-OE lines (AB29.2, AB29.7, and AB29.12) compared to controls (Additional file 5B, C). We also assessed leaf growth in the *PtGAUT12.1*-OE and control lines by comparing leaf areas of developing and fully expanded leaves, represented by the 10th and 20th leaf from the apex, respectively. Both developing and fully expanded leaf areas were significantly reduced by 68–74 and 70–74%, respectively, in all three OE lines examined compared to the controls (Additional file 5D, E). We then measured the relative water content (RWC), as described previously [5], of leaves from *PtGAUT12.1*-OE and control plants to determine whether there was a correlation between this parameter and the smaller leaf size in the OE lines. After 72 h, the RWC of leaves from *PtGAUT12.1*-OE lines was 6–12% lower than in leaves of WT (Additional file 5F). Comparison of relative water content and leaf size in the *PtGAUT12.1*-OE and *PdGAUT12.1*-KD lines indicated a positive correlation between these two parameters.

### *PtGAUT12.1* overexpression decreases the number of xylem cells and the size of xylem fiber and vessel cells in mature wood tissues

**Fig. 8:**
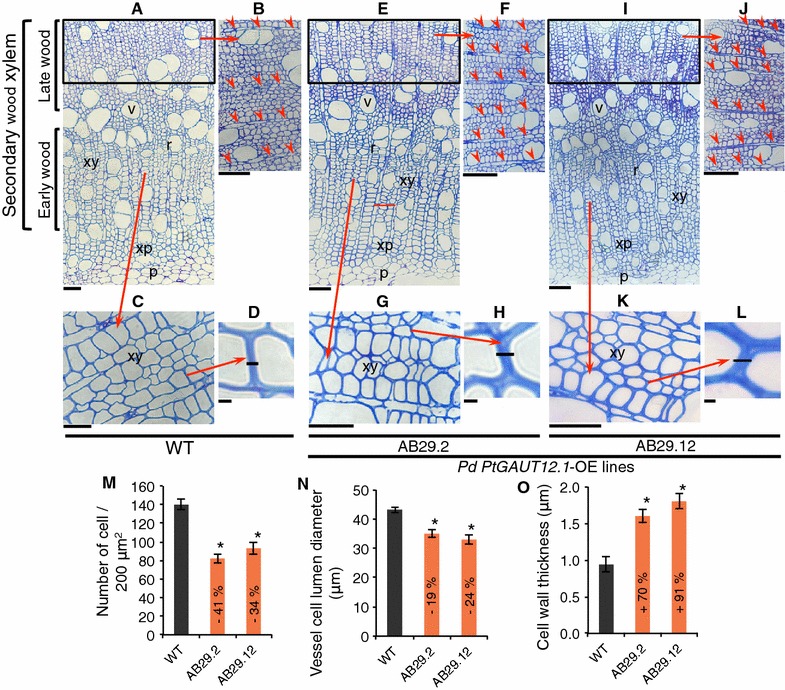
Microscopic analysis of stems from *P. deltoides* WT and *PtGAUT12.1*-OE lines. **A**–**L** Stem cross sections of the stem 20th internode from 3-month-old **A**–**D** WT and *PtGAUT12.1*-OE **E**–**H** AB29.2 and **I**–**L** AB29.12 lines. **B**, **F**, **J** Higher magnification of the late wood xylem from **A**, **E**, and **I**, respectively. Note that the transgenic lines appear to have more ray cells (marked with red arrow heads) within the same-sized area compared to WT. **C**, **G**, **K** Higher magnification of the early wood xylem from panels **A**, **E**, and **I**, respectively. **D**, **H**, **L** Higher magnification of the cell wall thickness from panels **C**, **G**, and **K**, respectively. **M** Number of individual xylem cells per 200 μm^2^ sub-areas within the area delineated by the black square on **a**, **E** and **I**. **n** Lumen diameter of xylem vessel cells of WT and transgenic lines. **O** Wall thickness of xylem cells of WT and transgenic lines. Error bars represent SD, *n* = 5, *P < 0.05. xy, xylem; r, xylem ray cells; xp, xylem parenchyma; v, xylem vessel; p, pith. Scale bars represent: **A**—70 μm; **B**—80 μm; **C**—30 μm; **D**, **H**, **L**—1 μm; **E**, **I**—100 μm; **F**, **J**—90 μm; **G**, **K**—50 μm

To examine the effect of *PtGAUT12.1* overexpression in secondary tissues, WT and *PtGAUT12.1*-OE 3-month-old plants were analyzed by microscopy of stem sections of the 20th internode from the top of stem. There was a significant 34–41% decrease in the number of late wood xylem fiber cells per 200 mm^2^ area in stem sections from the *PtGAUT12.1*-OE lines compared to WT (Fig. 8A, B, E, F, I, J, M), as well as a significant 19–24% decrease in the *PtGAUT12.1*-OE xylem vessel lumen diameter (Fig. 8A, E, I, N). Interestingly, we also observed a 70–91% increase in xylem fiber cell wall thickness of *PtGAUT12.1*-OE lines compared to WT (Fig. 8D, H, L, O).

**Fig. 9:**
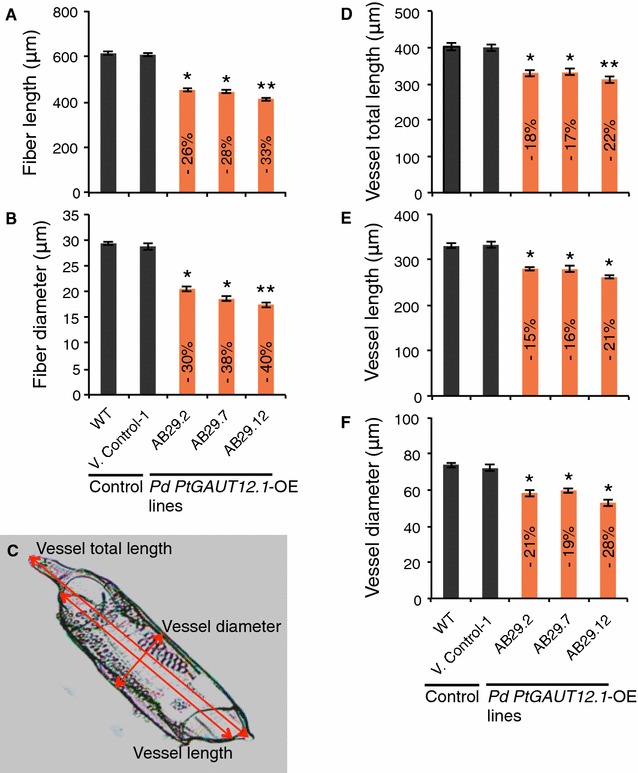
Xylem fiber and vessel cell size of *P. deltoides* WT and *PtGAUT12.1*-OE lines. **A**, **B** Xylem fiber length and diameter, respectively. **C** A vessel cell of *PtGAUT12.1*-OE line showing the parameters measured. **D**–**F** Xylem vessel total vessel length, lumen length, and lumen diameter, respectively. Transgenic values significantly different from wild type, as determined by ANOVA followed by Tukey’s multiple comparison test, are denoted with *(*P* < 0.05) or **(*P* < 0.001). *n* = 210

The size of individual wood cells isolated by maceration of debarked bottom stem from 9-month-old plants was also measured (as described previously [5]). Both fiber and vessel cells were smaller in *PtGAUT12.1*-OE plants compared to WT. Specifically, fiber cells of *PtGAUT12.1*-OE lines were 26–33% significantly shorter and had 30–40% reduced diameter compared to WT (Fig. 9A, B). Likewise, the vessel cells of *PtGAUT12.1*-OE lines were significantly smaller with 18–22% reduced total length, 15–21% reduced lumen length, and 19–28% reduced lumen diameter compared to WT vessel cells (Fig. 9C–F). These results indicate that reduced cell number and size in the stem may have led to the reduced plant height and stem diameter in the *PtGAUT12.1*-OE plants. The results also suggest that overexpression of *GAUT12.1* affects both the cell division and expansion in the secondary tissues.

### Xylose and galacturonic acid content are increased in *PtGAUT12.1*-OE walls

To determine the consequence of *PtGAUT12.1* overexpression on the non-cellulosic wall polysaccharides, we analyzed the glycosyl residue composition of wood from the three *PtGAUT12.1*-OE lines (AB29.2, AB29.7, and AB29.12) along with the WT and vector controls. The goal of these analyses was to test the two propositions regarding GAUT12 enzymatic function. If only xylose was increased in AIR from the *GAUT12* overexpression lines, this would support a function of GAUT12 in synthesizing the xylan reducing end sequence. Alternatively, if both xylose and GalA were increased, this would support a function for GAUT12 in synthesizing a pectic glycan required for xylan synthesis.

Wood cell walls were extracted as alcohol insoluble residue (AIR) from the bottom 6 cm of stems of greenhouse-grown, 9-month-old plants, and analyzed by gas chromatography–mass spectrometry (GC–MS) of trimethylsilyl (TMS) derivatives. The mol% of two sugars was significantly increased in AIR from *PtGAUT12.1*-OE lines compared to WT, i.e., 14–20% increased Xyl and 12–17% increased GalA (Table 1). There was also a non-significant trend for 19–35% increased Rha. A significantly greater amount of Xyl (21–28%) and GalA (16–23%) was also observed in the glycosyl residue composition of 2.8-year-old, field-grown trees of the same three OE lines compared to WT level (Fig. 7I, J, Additional file 6A), again confirming the stability of the phenotype of the *PtGAUT12.1*-OE lines in the field environment overtime. The increased Xyl and GalA content in the *PtGAUT12.1*-OE walls was accompanied by significantly decreased amounts of galactose (Gal), mannose (Man), and glucose (Glc), compared to controls (Table 1, Additional file 6A). The major effect of *PtGAUT12.1* overexpression on the mol% Xyl and GalA is in agreement with our previous study [5] supporting the role of GAUT12 in xylan and pectin biosynthesis in *P. deltoides*.

**Table 1:** Glycosyl residue composition of alcohol insoluble residue (AIR) from stems of greenhouse-grown, 9-month-old *P. deltoides* WT, vector control, and *PtGAUT12.1*-OE lines of tetramethylsilane (TMS) derivatives

	Glycosyl residue composition of AIR (mol% ± SE)								
	Ara	Rha	Fuc	Xyl	GlcA	GalA	Man	Gal	Glc
WT	3.06 ± 0.06	1.64 ± 0.06	0.09 ± 0.006	47.78 ± 1.21	0.21 ± 0.008	7.77 ± 0.21	6.08 ± 0.25	2.89 ± 0.14	30.48 ± 1.20
V Control-1	3.01 ± 0.08	1.79 ± 0.04	0.08 ± 0.004	47.26 ± 1.32	0.22 ± 0.006	6.97 ± 0.14	6.43 ± 0.28	3.01 ± 0.21	31.23 ± 1.32
AB29.2	2.96 ± 0.10	1.95 ± 0.06	0.07 ± 0.007	*54.28* *±* 1.01*	0.20 ± 0.010	*8.67* *±* 0.22*	*4.11* *±* 0.33*	*2.11* *±* 0.16*	*25.65* *±* 1.11*
AB29.7	2.88 ± 0.07	2.01 ± 0.05	0.10 ± 0.006	*55.64* *±* 1.11*	0.21 ± 0.005	*8.96* *±* 0.23*	*4.23* *±* 0.27*	*1.97* *±* 0.18*	*24.00* *±* 1.21*
AB29.12	2.57 ± 0.09	2.22 ± 0.08	0.08 ± 0.010	*57.43* *±* 0.98**	0.19 ± 0.007	*9.11* *±* 0.24**	*3.15* *±* 0.41**	*1.05* *±* 0.21**	*24.20* *±* 1.25*

The amounts of sugar are represented as average mol% of AIR ± SE of 3 biological and 2 technical replicates (*n* = 6). Italics numbers with stars indicate mutant values that are significantly different from WT at * P ≤ 0.05, ** *P* ≤ 0.001 significant level (one-way ANOVA followed by Tukey’s multiple comparison test)

### Analyses of fractionated cell walls from transgenic and WT biomass reveal reduced wall extractability, xylan, and HG in the *PtGAUT12.1*-OE lines

We reasoned that *PtGAUT12.1* overexpression might lead to the increased production of the GAUT12-synthesized polymer in a specific subfraction of wall material and thereby enable its purification and structural characterization. We therefore fractionated AIR samples from *PtGAUT12.1*-OE and control lines, from both greenhouse- and field-grown plants, by sequential extraction of AIR using increasingly harsh reagents. This technique yields a series of wall extracts enriched for specific classes of wall polymers [25, 26]. The wall extracts were analyzed for the amount of wall material recovered, the glycosyl residue composition and linkage, and the presence of specific carbohydrate epitopes via glycome profiling [25]. The goal was to identify a unique polymer(s) produced in the *PtGAUT12.1*-OE lines.

#### The amount of extractable cell wall material recovered from *PtGAUT12.1*-OE lines is reduced compared to WT

The yields of total AIR recovered from equivalent amounts of *PtGAUT12.1*-OE and control dry biomass were comparable (Additional file 7A). However, the amounts of wall material recovered in the sequential extracts of the AIR from *PtGAUT12.1*-OE lines were significantly less than those recovered from the controls. Specifically, the amounts of extractable wall material from the *PtGAUT12.1*-OE lines were decreased compared to WT by the following amounts in the designated extracts: ammonium oxalate extract (13–25%), sodium carbonate extract (23–43%), 1 M KOH extract (14–22%), 4 M KOH extract (15–22%), and 4 M KOH PC extract (9–19%). This resulted in a 9–15% decreased amount of total recoverable wall material from the combined *PtGAUT12.1*-OE extracts compared to the controls (Additional file 7B–E, G, H). The only exception was the sodium chlorite extract, for which comparable amounts of extract were recovered from AIR of the *PtGAUT12.1*-OE and control lines (Additional file 7F). Conversely, 8–15% more final insoluble pellet was recovered from the *PtGAUT12.1*-OE AIR compared to the WT lines (Additional file 7I). These results indicate that overexpression of *PtGAUT12.1* increases biomass recalcitrance, making it more difficult to extract the wall polymers from the *PtGAUT12.1*-OE lines than from the control plants. Since only two polymers were increased in abundance in the overexpression line, xylan and HG, the results supported one of the following three hypotheses: (1) increased xylan was inhibiting the ability to extract polymers from the wall, (2) increased HG was affecting wall extractability, or (3) an increased amount of a polymeric structure containing both xylan and HG was increased, restricting wall extractability.

#### Glycosyl residue composition analysis shows increased GalA and Xyl content in cell wall extracts from *PtGAUT12.1*-OE versus control lines

To test the three above hypotheses, the glycosyl residue composition of the different wall extracts was determined. Extraction of AIR using ammonium oxalate and sodium carbonate yields wall extracts enriched in pectic polymers, typified by the abundance of GalA (Table 2, Additional file 6B, C). For the samples from greenhouse-grown plants, the mol% GalA was significantly increased (by 12–19 and 21–36%, respectively) in both the ammonium oxalate and sodium carbonate extracts of the *PtGAUT12.1*-OE lines compared to WT (Table 2). Interestingly, the mol% Xyl in these wall extracts was also significantly increased (by 21–27 and 17–23%, respectively) in OE samples compared to the WT (Table 2). In the ammonium oxalate extracts, the greater mol% GalA and Xyl was accompanied by slightly increased mol% Rha and Gal in the *PtGAUT12.1*-OE samples. GalA and Xyl were also increased in the sodium carbonate extracts. Similar trends were found in the field-grown plants (Additional file 6B, C). These results suggested that the two pectin-enriched extracts of the *PtGAUT12.1*-OE lines contained increased pectin and xylan content.

**Table 2:** Glycosyl residue composition of cell wall fractions from stem of *P. deltoides* WT, vector control, and *PtGAUT12.1*-OE plants

	Glycosyl residue composition of cell wall fractions (mol% ± SE)								
	Ara	Rha	Fuc	Xyl	GlcA	GalA	Man	Gal	Glc
Ammonium oxalate									
WT	19.5 ± 0.8	5.1 ± 0.2	0.7 ± 0.02	8.4 ± 0.3	0.7 ± 0.01	23.6 ± 0.6	11.8 ± 0.7	4.5 ± 0.2	25.7 ± 1.3
V Control-1	18.9 ± 0.9	4.8 ± 0.1	0.7 ± 0.03	8.5 ± 0.3	0.7 ± 0.01	23.4 ± 0.7	12.6 ± 0.6	4.1 ± 0.2	26.3 ± 1.2
AB29.2	17.6 ± 0.9	5.3 ± 0.2	0.6 ± 0.02	*10.2* *±* 0.2*	0.7 ± 0.01	*26.5* *±* 0.8*	11.9 ± 0.4	*5.1* *±* 0.1*	*22.5* *±* 1.1*
AB29.7	18.1 ± 0.5	*5.6* *±* 0.1*	0.6 ± 0.02	*10.5* *±* 0.3*	0.6 ± 0.01	*27.4* *±* 0.9*	12.1 ± 0.6	4.8 ± 0.2	*20.2* *±* 0.9*
AB29.12	17.9 ± 0.6	5.4 ± 0.2	0.7 ± 0.03	*10.7* *±* 0.4**	0.7 ± 0.01	*28.1* *±* 0.5*	12.2 ± 0.5	4.7 ± 0.1	*19.6* *±* 1.1*
Sodium carbonate									
WT	11.3 ± 0.6	5.8 ± 0.2	0.3 ± 0.03	22.4 ± 0.5	1.6 ± 0.06	18.9 ± 0.5	15.1 ± 0.6	6.8 ± 0.2	17.8 ± 0.8
V Control-1	11.4 ± 0.5	6.0 ± 0.3	0.2 ± 0.03	22.3 ± 0.5	1.3 ± 0.05	18.7 ± 0.6	15.3 ± 0.5	6.5 ± 0.3	18.3 ± 0.7
AB29.2	*8.3* *±* 0.5*	5.1 ± 0.3	0.4 ± 0.04	*26.3* *±* 0.3*	2.3 ± 0.06	*22.9* *±* 0.4*	14.6 ± 0.4	*5.2* *±* 0.2*	*14.9* *±* 0.6*
AB29.7	*8.1* *±* 0.4*	5.5 ± 0.2	0.3 ± 0.03	*26.9* *±* 0.4*	2.4 ± 0.05	*23.6* *±* 0.7*	14.1 ± 0.6	*4.9* *±* 0.2*	*14.2* *±* 0.7*
AB29.12	*7.8* *±* 0.6*	5.4 ± 0.3	0.5 ± 0.02	*27.6* *±* 0.6*	2.5 ± 0.06	*25.7* *±* 0.6*	*13.5* *±* 0.5*	*4.7* *±* 0.3*	*12.3* *±* 0.8*
1 M KOH									
WT	0.3 ± 0.01	3.7 ± 0.1	0.2 ± 0.01	69.9 ± 1.3	8.7 ± 0.2	3.2 ± 0.1	5.9 ± 0.2	1.4 ± 0.08	6.7 ± 0.2
V Control-1	0.2 ± 0.01	3.5 ± 0.1	0.2 ± 0.01	70.0 ± 1.5	8.7 ± 0.3	3.1 ± 0.1	6.0 ± 0.3	1.6 ± 0.05	6.7 ± 0.4
AB29.2	0.3 ± 0.02	*1.1* *±* 0.1*	0.3 ± 0.01	*79.8* *±* 2.1*	*9.9* *±* 0.1*	*3.6* *±* 0.05*	*2.3* *±* 0.3*	*0.9* *±* 0.06**	*1.8* *±* 0.3**
AB29.7	0.2 ± 0.01	*1.2* *±* 0.3*	0.2 ± 0.01	*81.1* *±* 2.7*	*9.9* *±* 0.2*	*3.7* *±* 0.06*	*1.1* *±* 0.2*	*0.6* *±* 0.09**	*2.0* *±* 0.4**
AB29.12	0.2 ± 0.01	*0.7* *±* 0.3*	0.1 ± 0.01	*82.2* *±* 2.2**	*10.0* *±* 0.2*	*3.9* *±* 0.06**	*1.2* *±* 0.4*	*0.2* *±* 0.06**	*1.5* *±* 0.5**
4 M KOH									
WT	2.1 ± 0.09	3.2 ± 0.1	0.3 ± 0.02	39.1 ± 1.4	4.7 ± 0.2	0.9 ± 0.03	10.8 ± 0.5	6.6 ± 0.2	32.3 ± 1.6
V Control-1	2.1 ± 0.03	3.1 ± 0.1	0.4 ± 0.02	39.0 ± 1.1	4.8 ± 0.1	0.9 ± 0.03	11.0 ± 0.3	6.9 ± 0.3	31.8 ± 1.7
AB29.2	2.2 ± 0.06	3.6 ± 0.08	0.5 ± 0.02	*46.7* *±* 1.3*	*5.7* *±* 0.2*	*1.2* *±* 0.05*	*10.5* *±* 0.6**	*4.9* *±* *0.4*	*24.7* *±* 1.5*
AB29.7	1.8 ± 0.05	3.4 ± 0.1	0.4 ± 0.01	*44.3* *±* 1.1*	*5.4* *±* 0.1*	*1.1* *±* 0.03*	*9.6* *±* 0.3*	*5.8* *±* 0.6**	*28.2* *±* 1.4*
AB29.12	2.0 ± 0.06	*3.8* *±* 0.09*	0.3 ± 0.01	*49.8* *±* 1.5**	*6.1* *±* 0.2*	*1.2* *±* 0.03**	*8.7* *±* 0.5**	*4.8* *±* 0.3*	*23.3* *±* 1.6**
Sodium chlorite									
WT	6.7 ± 0.3	3.9 ± 0.2	0.1 ± 0.01	11.6 ± 0.4	0.9 ± 0.03	7.8 ± 0.2	2.0 ± 0.04	11.7 ± 0.3	55.4 ± 1.5
V Control-1	6.9 ± 0.4	4.1 ± 0.2	0.2 ± 0.01	11.4 ± 0.6	0.8 ± 0.04	6.9 ± 0.2	1.9 ± 0.06	11.8 ± 0.4	56.0 ± 1.4
AB29.2	*5.8* *±* 0.4*	*5.1* *±* 0.1*	–	*14.5* *±* 0.4*	1.1 ± 0.03	*8.9* *±* 0.2*	*1.7* *±* 0.08*	11.6 ± 0.5	51.3 ± 1.1
AB29.7	*6.1* *±* 0.2*	*4.3* *±* 0.1*	–	*13.3* *±* 0.3*	1.0 ± 0.02	*8.6* *±* 0.1*	*1.5* *±* 0.09*	12.3 ± 0.4	52.9 ± 1.3
AB29.12	*6.2* *±* 0.3*	*5.0* *±* 0.2*	–	*15.4* *±* 0.5**	1.3 ± 0.05	*10.1* *±* 0.3*	*1.4* *±* 0.1**	11.1 ± 0.6	*49.5* *±* 0.9*
4 M KOH PC									
WT	0.9 ± 0.03	2.2 ± 0.06	0	66.4 ± 1.9	8.2 ± 0.3	2.1 ± 0.09	6.0 ± 0.2	4.7 ± 0.3	9.5 ± 0.6
V Control-1	0.9 ± 0.05	2.3 ± 0.07	0	66.0 ± 2.6	8.1 ± 0.4	2.0 ± 0.10	5.9 ± 0.3	5.0 ± 0.3	9.8 ± 0.3
AB29.2	1.0 ± 0.03	*2.5* *±* 0.06*	0	*78.3* *±* 1.3*	*9.6* *±* 0.3*	*2.7* *±* 0.04*	*1.9* *±* 0.3**	*1.3* *±* 0.2*	*2.7* *±* 0.3**
AB29.7	0.9 ± 0.04	*2.4* *±* 0.08*	0	*75.1* *±* 1.2*	*9.4* *±* 0.2*	*2.5* *±* 0.05*	*3.5* *±* 0.2*	*1.6* *±* 0.3**	*4.6* *±* 0.4*
AB29.12	0.6 ± 0.06	2.3 ± 0.07	0	*80.8* *±* 1.5**	*9.8* *±* 0.4**	*2.8* *±* 0.07**	*1.0* *±* 0.4**	*0.8* *±* 0.2**	*1.9* *±* 0.2**
Insoluble									
WT	3.9 ± 0.3	2.8 ± 0.1	0.2 ± 0.02	14.9 ± 0.3	0.2 ± 0.03	3.0 ± 0.4	9.5 ± 0.3	5.6 ± 0.1	59.9 ± 0.9
V Control-1	4.0 ± 0.1	3.1 ± 0.1	0.3 ± 0.03	15.3 ± 0.2	0.2 ± 0.04	2.9 ± 0.6	9.9 ± 0.2	5.4 ± 0.3	58.9 ± 0.8
AB29.2	3.9 ± 0.3	*3.5* *±* 0.1**	0.2 ± 0.02	*16.0* *±* 0.4*	0.2 ± 0.02	*4.9* *±* 0.3**	*5.9* *±* 0.3**	*1.8* *±* 0.2**	63.6 ± 0.7
AB29.7	3.8 ± 0.3	*3.8* *±* 0.09**	0.3 ± 0.02	*16.4* *±* 0.3*	0.3 ± 0.01	*4.7* *±* 0.5**	*4.7* *±* 0.4**	*1.7* *±* 0.3**	*64.3* *±* 0.6*
AB29.12	3.8 ± 0.3	*4.1* *±* 0.1**	0.3 ± 0.03	*17.0* *±* 0.5*	0.2 ± 0.02	*5.5* *±* 0.4**	*3.5* *±* 0.4**	*1.4* *±* 0.2**	*64.2* *±* 0.7*

Alcohol insoluble residue (AIR) was sequentially extracted using ammonium oxalate, sodium carbonate, 1 M KOH, 4 M KOH, sodium chlorite (chlorite), and 4 M KOH post-chlorite (PC). Each fraction was then analyzed by GC–MS of tetramethylsilane (TMS) derivatives. The amounts of sugar are average mol% of wall extracts ± SE of 3 biological and 2 technical replicates (*n* = 6). Italics numbers with stars are mutant values that are significantly different from WT at * *P* ≤ 0.05, ** *P* ≤ 0.001 significant level (one-way ANOVA followed by Tukey’s multiple comparison test). A dash indicates that the sugar level was below detection limits

Fractionation of the remaining AIR with the alkaline solvents 1 M KOH and 4 M KOH extracted cell wall material enriched in hemicellulosic polysaccharides, as indicated by the large amounts of Xyl (Table 2, Additional file 6D, E). The 1 M and 4 M KOH extracts of greenhouse-grown *PtGAUT12.1*-OE lines were significantly increased, compared to WT, respectively, by 14–18 and 13–27% mol% Xyl, and 13–22 and 22–33 mol% GalA, respectively (Table 2). A 14–15 and 15–30% increase in mol% GlcA was also observed in 1 M and 4 M KOH extracts, respectively, from *PtGAUT12.1*-OE lines compared to controls (Table 2). In contrast, the mol% Man, Gal, and Glc were noticeably decreased in both extracts, as was Rha in the 1 M KOH extract of *PtGAUT12.1*-OE lines compared to controls (Table 2). The same trends were observed in samples from field-grown plants (Additional file 6D, E). The results suggest that *PtGAUT12.1* overexpression substantially increases the amount of (glucurono)xylan in *PtGAUT12.1*-OE walls but also affects lesser amounts of pectic polymers present in these fractions.

The insoluble wall material remaining after the 4 M KOH extraction step was further treated with sodium chlorite to release polymers ostensibly held into the wall by association with lignin (Table 2, Additional file 6F). For the samples from greenhouse-grown plants, Rha, Xyl, and GalA were, respectively, significantly increased by 10–31, 15–33, and 10–30% in the *PtGAUT12.1*-OE chlorite extracts, while Man was markedly decreased compared to controls (Table 2). The final post-chlorite (PC) 4 M KOH extraction step yielded a Xyl-rich extract (Table 2, Additional file 6G). The 4 M KOH PC extracts from greenhouse-grown *PtGAUT12.1*-OE lines had substantially 13–22% increased mol% Xyl, 15–20% increased GlcA, and 19–33% increased GalA compared to controls (Table 2) and significantly decreased mol% Man, Gal, and Glc compared to controls. Lastly, the final insoluble pellets remaining after all extraction steps were analyzed for sugar composition (Table 2, Additional file 6H). The greatest increase in the greenhouse *PtGAUT12.1*-OE samples over WT was 63–85% increased mol% GalA, followed by 25–46% increased Rha, and 7–14% increased Xyl along with a small 6–7% increase in mol% Glc (Table 2). These increases were accompanied by a substantial 38–63% decrease in mol% Man and 68–75% decrease in Gal (Table 2). Similar mol% increase/decrease values were observed in extracts from field-grown plants, indicating that the results were sustained in the field-grown lines (Additional file 6F–H).

The mol% sugar composition data provide a facile means to compare the relative abundance of the different monosaccharides in total AIR or AIR extracts isolated from transgenic and control lines. Mass yield data, on the other hand, provide information on the actual amounts of the different sugars present in the cell wall samples [27]. We thus also analyzed the μg yield of each sugar per mg AIR for each of the cell wall extracts (Additional file 8). In general, the mol% and mass yield data showed similar general trends. For example, in both data formats, Xyl and GalA are the only sugar residues whose amounts increased across all wall fractions, including the insoluble pellets. However, there were some minor exceptions. For example, in the *PtGAUT12.1*-OE samples compared to WT, there was an increased mol% of GlcA in both the 1 M KOH and 4 M KOH extracts, and an increased mol% of Rha in the 4 M KOH PC extract. However, the mass yield data showed a decrease in the total amount of these sugars per mg AIR due to the reduced amount of the 1 M KOH, 4 M KOH, and 4MKOHPC fractions in the *PtGAUT12.1*-OE samples compared to WT (compare Table 2 and Additional file 8). Overall, the data are consistent with *GAUT12.1* having a role in the biosynthesis of HG and xylan in *P. deltoides* and best support hypothesis 3, i.e., that GAUT12.1 is involved in the synthesis of a polymer containing both xylan and HG.

It is interesting to note that analysis of the final pellets remaining after all extractions of AIR from the WT, *PtGAUT12*-OE, and *PdGAUT12*-KD lines revealed, surprisingly, that the final pellets contained a greater amount of GalA than any of the extracts (Additional files 8, 9). Furthermore, the final pellets from the *PtGAUT12*-OE lines also had the largest increased amount of GalA compared to any of the extracts. This result suggests that poplar *GAUT12.1* may function in the synthesis of an HG-containing structure that is part of a foundational cell wall architecture held tightly in the wall and required for the synthesis of xylan.

#### Glycosyl linkage analysis of *PtGAUT12.1*-OE cell wall fractions confirms effects on xylan and pectin

To confirm whether the increased Xyl and GalA contents were indeed associated with xylan and HG, we compared the glycosyl residue linkages of wall carbohydrates recovered in the ammonium oxalate, sodium carbonate, 1 M KOH wall extracts, and insoluble pellets from AIR of greenhouse-grown *PtGAUT12.1*-OE lines AB29.2 and AB29.12 and WT (Table 3, Additional file 10). In the ammonium oxalate extracts (Table 3), the greater GalA content in the *PtGAUT12.1*-OE lines compared to controls was due to a 3–3.7 mol% increase in 4-linked GalA*p* and a 0.8 mol% increase in terminal-GalA*p.* The 2-linked Rha*p*, a constituent of the RG-I backbone, was increased 0.2–0.3 mol% in these same extracts. The results confirm a higher accumulation of HG accompanied by a smaller increase in RG-I in the cell wall upon *PtGAUT12.1* overexpression. Likewise, the increased amount of xylan in the *PtGAUT12.1*-OE ammonium oxalate extracts was confirmed by the 1.1–1.4 mol% increased 4-linked Xyl*p* compared to WT in these samples.

**Table 3:** Glycosyl linkage analysis of cell wall fractions from stems of *P. deltoides* WT and *PtGAUT12.1*-OE lines

	Ammonium oxalate soluble			Sodium carbonate soluble			1 M KOH soluble		
	WT	AB29.2	AB29.12	WT	AB29.2	AB29.12	WT	AB29.2	AB29.12
t-Ara*f*	3.6	3.7	3.7	3.5	3.1	2.5	0.2	0.2	0.2
t-Ara*p*	0.2	0.2	0.2	0.2	0.2	0.2	0.1	0.1	0.1
3-Ara*f*	0.5	0.7	0.8	0.5	0.4	0.4	–	–	–
4-Ara*p* or 5-Ara*f*	3.2	1.9	1.8	2.1	1.0	1.1	0.2	0.1	1.1
3,4-Ara*p* or 3,5-Ara*f*	1.5	–	1.5	–	–	–	–	–	–
t-Rha*p*	0.9	0.9	0.9	1.9	1.1	1.3	0.2	0.2	0.2
2-Rha*p*	2.2	2.4	2.5	2.9	3.1	3.3	0.7	0.4	1.1
3-Rha*p*	–	–	–	–	–	–	1.6	2.0	2.2
2,4-Rha*p*	1.1	0.9	1.3	1.1	1.3	1.4	0.5	0.3	0.2
t-Fuc*p*	0.3	0.5	0.3	0.3	0.4	0.8	0.2	0.3	0.2
t-Xyl*p*	0.4	0.6	0.7	1.5	1.8	1.9	0.8	0.9	0.9
4-Xyl*p*	6.5	7.9	7.6	20.1	23.7	24.2	57.8	66.5	69.4
2,4-Xyl*p*	0.9	1.0	1.1	1.5	1.7	1.8	8.8	5.4	3.5
t-Man*p*	1.2	1.7	1.6	1.8	1.4	1.0	–	–	–
4-Man*p*	17.1	18.5	17.2	19.6	18.8	17.3	6.4	4.7	2.9
4,6-Man*p*	0.7	–	0.3	–	–	–	–	–	–
t-GlcA*p*	–	–	–	1.8	2.7	2.9	7.5	8.3	8.6
2-GlcA*p*	0.1	0.1	0.1	0.2	0.3	0.4	–	–	–
t-GalA*p*	1.1	1.9	1.9	2.0	3.1	3.3	–	1.1	1.3
2-GalA*p*	–	–	–	0.9	1.2	1.3	1.8	1.9	2.1
4-GalA*p*	23.8	26.8	27.5	16.1	19.9	20.8	1.5	4.7	4.9
3,4-GalA*p*	–	–	–	0.4	0.7	0.8	–	–	–
2,4-GalA*p*	0.1	0.3	0.4	0.1	–	–	–	–	–
t-Gal*p*	2.9	2.6	2.8	3.2	2.1	2.0	1.2	0.3	0.1
2-Gal*p*	0.4	–	0.4	0.6	–	–	–	–	–
4-Gal*p*	–	–	–	–	–	–	–	–	–
6-Gal*p*	0.4	0.7	0.4	–	–	–	0.5	0.6	0.2
3,4-Gal*p*	–	1.7	0.4	0.3	–	–	–	–	–
2,4-Gal*p*	0.2	0.2	0.2	0.2	–	–	–	–	–
t-Glc*p*	3.3	3.6	3.9	3.6	1.9	2.5	–	–	–
2-Glc*p*	0.3	0.1	0.2	0.1	0.2	0.1	–	–	–
4-Glc*p*	26.7	21.1	20.0	13.5	9.9	8.7	9.8	2.0	0.8
4,6-Glc*p*	0.4	–	0.6	–	–	–	–	–	–

All numbers are mol percentages. A dash designates sugar level below detection limits. Glycosyl linkage analysis data were provided by CCRC Analytical Services

The sodium carbonate extracts from *PtGAUT12.1*-OE had increased mol% amounts of 4-GalA*p* (3.8–4.7), 2-GalA*p* (0.3–0.4), and terminal-GalA*p* (1.1–1.3) in comparison to controls (Table 3). Although the sugar composition of this wall fraction did not indicate increased amount of Rha (Table 2), the 2-linked Rha*p* and 2,4-Rha*p* were increased 0.2–0.4 and 0.2–0.3 mol%, respectively, in *PtGAUT12.1*-OE samples (Table 3). Similarly, we observed 3.6–4.1 increased mol% 4-Xyl*p*, as well as 0.3–0.4 terminal-Xyl*p*, 0.2–0.3 2,4-Xyl*p*, and 0.9–1.1 t-GlcA*p* mol% increases in the sodium carbonate extracts of *PtGAUT12.1*-OE compared to WT (Table 3). These data again are consistent with greater quantities of HG and xylan due to the *PtGAUT12.1* overexpression.

Following a similar trend as observed in the ammonium oxalate and sodium carbonate AIR extracts, the *PtGAUT12.1*-OE 1 M KOH extracts were increased in sugar linkages characteristic of xylan and HG (Table 3). Compared to WT, the *PtGAUT12.1*-OE samples had 8.7–11.6 mol% increased for 4-Xyl*p*, 0.1 mol% increased t-Xyl*p*, and 0.8–1.1 mol% increased t-GlcA*p,* suggesting increased amounts of (glucurono)xylan. This was accompanied by a 0.1–0.3 mol% increased 2-GalA*p* and 0.4–0.6 mol% increased 3-Rha*p* in the *PtGAUT12.1*-OE samples compared to WT, suggesting a concurrent increase of the xylan reducing end sequence. The *PtGAUT12.1*-OE 1 M KOH extracts also had 3.2–3.4 mol% increased 4-GalA*p* and at least 1.1–1.3 mol% t-GalA*p*, consistent with an increased amount of HG.

Interestingly, glycosyl linkage analysis of the final insoluble pellets from the WT and *PtGAUT12*-OE lines identified fewer types of sugar linkages (Additional file 10) than in the soluble wall extracts (Table 3). Only seven glycosyl linkages were increased in the insoluble pellets of the *PtGAUT12.1*-OE lines compared to WT. Sugar linkages associated with pectin HG and RG-I backbones, 4-GalA*p*, t-GalA*p*, and 2,4-Rha*p*, were increased by up to 0.5, 0.3, and 0.2 mol%, and sugar linkages associated with xylan, 4-Xyl*p*, t-GlcA*p*, were increased by 0.2 and 0.1 mol%, respectively, in *PtGAUT12.1*-OE insoluble pellets compared to WT. The only other glycosyl linkages increased in the final pellets of *PtGAUT12.1*-OE lines compared to WT were 4-Man*p* and t-Glc*p* which were increased by 5.1 and 0.4 mol%, respectively. Intriguingly, analysis of the final pellets remaining in the *PdGAUT12.1*-KD lines (Additional files 10) also revealed the same limited types of glycosyl linkages, and of those only nine glycosyl linkages were decreased in the insoluble pellets of *PdGAUT12.1*-KD lines compared to WT. Sugar linkages associated with pectin HG and RG-I backbones, 4-GalA*p*, t-GalA*p*, and 2,4-Rha*p*, were decreased by up to 0.8, 0.2, and 0.2 mol%, respectively, and sugar linkages associated with xylan, 4-Xyl*p*, t-GlcA*p*, were decreased by 0.3 and 0.2 mol%, respectively, in the *PdGAUT12.1*-KD insoluble pellets compared to WT. The other glycosyl linkages decreased in the final pellets of the *PtGAUT12.1*-OE lines compared to WT were 4-Man*p*, 4,6-Man*p*, t-Glc*p*, and 3,4-Glc*p* which were decreased by 3, 0.1, 0.4, and 0.1 mol%, respectively. Taken together, the glycosyl linkage data provide support for the hypothesis that poplar *GAUT12.1* is involved in the synthesis of an HG-containing glycan that contains an RG-I, RG-I/xylan (see the Arabinoxylan Pectin Arabinogalactan Protein1 (APAP1) proteoglycan structure in [28]) or novel pectin-xylan structure required for the synthesis of (glucurono)xylan synthesis.

#### Glycome profiling reveals increased binding of specific xylan and HG backbone antibodies in certain wall fractions of *PtGAUT12.1*-OE compared to control

To further investigate the types of wall glycans increased in the *PtGAUT12.1*-OE lines, and antibodies that may recognize these structures, we subjected the sequentially extracted wall fractions from *PtGAUT12.1*-OE and WT lines to glycome profiling analyses. A set of 155 monoclonal antibodies (mAbs) raised against diverse non-cellulosic plant cell wall polysaccharides and reactive to different non-cellulosic glycan epitopes [29] was used to screen the wall extracts in this ELISA-based assay [25], with the goal of obtaining information about the presence and relative abundance of specific epitopes that are characteristic of different types of non-cellulosic polymers in each extract. The binding strength of each mAb across the different wall extracts and plant lines was visualized as heat maps [25].

**Fig. 10:**
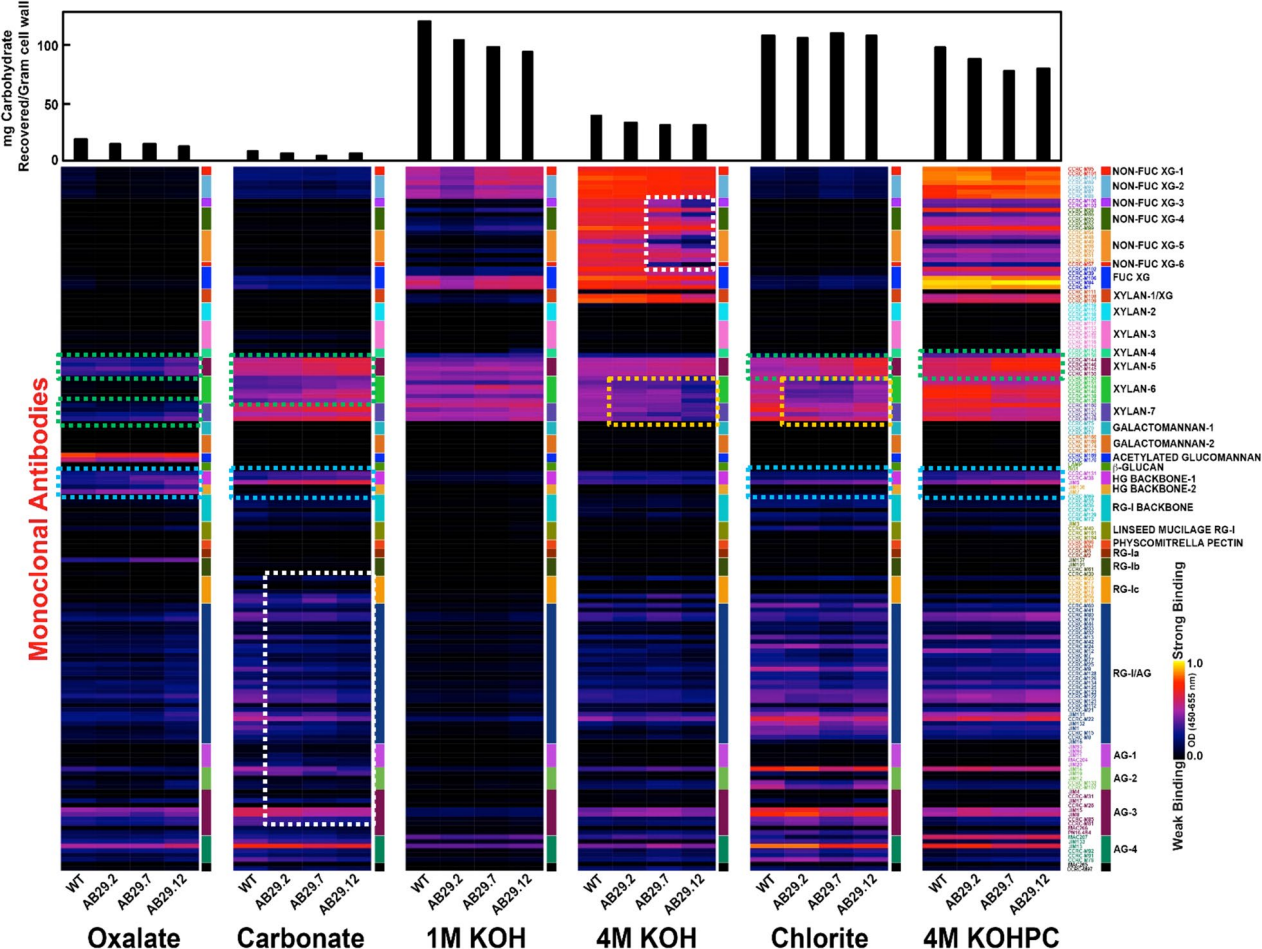
Glycome profiling of *P. deltoides PtGAUT12.1*-OE lines. Cell walls (AIR) prepared from WT and *PtGAUT12.1*-OE lines **(**AB29.2, AB29.7, and AB29.12) were sequentially extracted using increasingly harsh reagents as described in “Methods.” The resulting wall extracts were screened by ELISA using 155 monoclonal antibodies (mAbs) directed against epitopes present on most major non-cellulosic plant cell wall glycans [25, 29]. The binding response data are presented as heatmaps using a white-red-dark-blue scale indicating the strength of the ELISA signal (white, red, and dark-blue colors depict strong, medium, and no binding, respectively). The mAbs are grouped based on the cell wall glycans they predominantly recognize as depicted in the panel on right-hand side of the figure. The gravimetric amounts of materials extracted from the wall by each extracting reagent are shown as bar graphs above the heatmaps. Dotted boxes show major regions of the profiles where increased/reduced antibody binding was observed in *PtGAUT12.1*-OE wall extracts

The glycome profiling data (Fig. 10) showed both increases and decreases in epitope contents in the *PtGAUT12.1*-OE cell wall extracts compared to WT. The most consistent changes across multiple extracts were observed for xylan backbone epitopes recognized by the Xylan-6 and Xylan-7 groups of mAbs [30, 31], 4-*O*-methyl-GlcA-substituted xylans recognized by the Xylan-5 mAbs [30], and the epitopes recognized by HG backbone-specific mAbs [32]. Increased binding of mAbs that specifically bind to the 4-*O*-methyl GlcA side chains of xylans was observed in the oxalate, carbonate, chlorite, and 4 M KOHPC extracts of *PtGAUT12.1*-OE compared to WT (green boxes, Fig. 10). In addition, increased binding of CCRC-M150, which is specific for GlcA-substituted xylan [30], was observed in the 4 M KOHPC extract. No binding of CCRC-M154, a mAb specific for arabinosyl-substituted xylans [31], was observed in any cell wall extract from either OE or WT lines. Decreased binding of xylan backbone-directed mAbs (Xylan-6 and Xylan-7) was observed in the 4 M KOH, chlorite, (orange boxes, Fig. 10) and to a lesser extent, in the 4 M KOH PC extracts. For HG polymers, there was increased binding of mAbs directed against the de-methylesterified (HG backbone-1) and methylesterified (HG backbone-2) HG backbones in the oxalate extracts from *PtGAUT12.1*-OE AIR compared to WT, while in the other wall extracts (carbonate, chlorite, and 4 M KOHPC) there was increased binding of only the de-methylesterified HG backbone-directed mAbs in the OE lines (blue boxes, Fig. 10). In contrast, decreased binding was evident for mAbs in the RG-I/AG and AG-2, -3, and -4 groups in carbonate extracts and for non-fucosylated xyloglucan groups 3-6 in 4 M KOH extracts of the *PtGAUT12.1*-OE lines compared to WT (white boxes, Fig. 10). Overall, the glycome profiling data demonstrate altered wall polymer extractability as a result of *GAUT12.1* overexpression in poplar, with a trend towards more recalcitrant biomass from which it becomes harder to extract wall polymers.

## Discussion

We have overexpressed *PtGAUT12.1*, the higher expresser of the two poplar orthologs of Arabidopsis *GAUT12*, in *P. deltoides*. The generated poplar transgenics had 7–49% increased total *GAUT12.1* (both the introduced *PtGAUT12.1* transgene and the endogenous *PdGAUT12.1*) transcript expression level, leading to a 4–12% reduced saccharification yield for greenhouse-grown transgenic biomass. Plant growth was also negatively affected in *PtGAUT12.1*-OE lines, with 6–54% reduced plant height, 8–41% reduced radial stem diameter and, most importantly, 48–61% reduced dry biomass yield compared to controls. The elevated transcript level, increased recalcitrance, and decreased growth were notably maintained in *PtGAUT12.1*-OE plants grown for more than 2 years in the field, demonstrating the transgene stability in the environment over time. Most importantly, *PtGAUT12.1*-OE plants displayed exactly the opposite phenotypes of *PdGAUT12.1*-KD (knockdown) plants described in our previous research [5], in which downregulation of *PdGAUT12.1* by RNA silencing resulted in significantly increased saccharification efficiency and improved growth. Furthermore, the opposing phenotypes of the *PtGAUT12.1*-OE versus *PdGAUT12.1*-KD lines extend to almost all parameters measured in this work, including leaf phenotypes, cell size, and extractability, sugar composition, and sugar linkages of the cell wall. Overall, the combined OE and KD data clearly establish *GAUT12.1* as a recalcitrance- and growth-associated gene in poplar.

The phenotypes of the *PtGAUT12.1*-OE plants verified our previous results [5] showing that modifying *GAUT12* expression in poplar yields outcomes different than in Arabidopsis. The knocking-out of Arabidopsis *GAUT12/IRX8* resulted in reduced wall xylan content, a collapsed xylem phenotype, and dwarfed *irx8* mutant plants [13, 14], while overexpression of the gene did not alter wall composition or plant growth [15]. We showed previously that *PdGAUT12.1* could complement the phenotype of Arabidopsis *irx8* mutants, indicating that it is a functional ortholog of the Arabidopsis *GAUT12* [5]. However, silencing of *PdGAUT12.1* in poplar, while also causing significantly reduced xylan content, resulted in increased saccharification without compromising growth; rather it increased plant growth [5]. The increased xylan content and reduced plant growth of the *PtGAUT12.1*-OE plants, thus confirmed that the effects of modifying expression of a gene in Arabidopsis does not necessarily translate to woody plants and, as such, highlights the need for phenotypes to be verified across species.

The reduced plant growth and biomass yield of the *PtGAUT12.1*-OE lines compared to controls may, at least partially, be due to the decreased leaf size, xylem vessel cell size, and relative water content. Since water flow is smaller in smaller radius vessels [33], the reduced cell size of xylem vessel cells could explain the reduced water content and poorer growth of *PtGAUT12.1*-OE compared to WT. In contrast, *PdGAUT12.1*-KD lines had the reverse characteristics [5] with increased cell size, water content, and plant growth. Such effects of modified *GAUT12.1* expression on cell size, water content, and wall integrity suggest that the mechanism(s) by which modified *GAUT12* expression leads to modified cell and plant growth may be complex and multifactorial. Previous studies of GAUT12 have focused heavily on the stem due to the high *GAUT12* transcript expression in this tissue. However, weaker expression of *GAUT12* has also been reported in the vascular tissues of leaves and petioles of Arabidopsis and poplar [5, 11, 14, 22, 34]. We show here that weak expression of poplar *GAUT12*, especially *PdGAUT12.1*, indeed is detectable in *P. deltoides* developing organs, i.e., apex, young leaves, and young internodes (Fig. 2C, D). This suggests that at least some effects of modifying *GAUT12.1* expression may already have taken place in young tissues and may explain the reduction and increase in the overall growth of *PtGAUT12.1*-OE and *PdGAUT12.1*-KD plants, respectively, as opposed to phenotypes occurring only in the secondary wall-rich stem. The *PtGAUT12.1*-OE growth phenotype, however, may also be due to a plant-wide effect as a result of the use of *A. thaliana Ubiquitin3* promoter to drive the overexpression of *PtGAUT12.1* constitutively in *P. deltoides*.

Cell wall sugar composition analyses revealed substantially increased Xyl and GalA content in the total AIR and also in virtually every single wall fraction extracted from AIR of the *PtGAUT12.1*-OE biomass compared to controls, both from greenhouse- and field-grown plants (Tables 1, 2, Additional files 6, 8). Together with the sugar linkage data (Table 3, Additional file 10), these data indicated increased amounts of xylan and HG as a result of *PtGAUT12.1* overexpression, and were consistent with the hypothesis that *GAUT12* encodes a GT that synthesizes a wall structure containing, or required for the formation of, both xylan and HG in poplar wood.

Analysis of the sugar composition, linkage, and glycome profiling data of *P. deltoides PdGAUT12.1*-KD (Additional files 9, 10, and in [5]) and *PtGAUT12.1*-OE (Tables 1, 2, 3, Additional files 8, 10, Fig. 10) biomass revealed several interesting observations. First, both Xyl and GalA contents were increased in all *PtGAUT12.1*-OE wall fractions compared to controls (Table 2, Additional file 8). However, only GalA content was reciprocally decreased in every single wall extract of the *PdGAUT12.1*-KD biomass (Additional file 9, [5]). Xyl content was indeed reduced in the total AIR and in most of the wall extracts of the *PdGAUT12.1*-KD biomass, yet it was increased in the first two wall fractions, i.e., the ammonium oxalate and sodium carbonate extracts (Additional files 9, [5]). These results suggest that knockdown of *PdGAUT12.1* led to a subfraction of xylan that was more easily extracted from the wall. These changes in GalA content, rather than in Xyl, in all wall fractions suggest that *PdGAUT12.1* functions directly in the synthesis of a specific HG glycan required for xylan synthesis, rather than directly in the synthesis of xylan itself.

Secondly, it is noteworthy that a substantial portion of GalA actually resides in the insoluble pellets of both the *PdGAUT12.1*-KD and *PtGAUT12.1*-OE cell walls that remain after all the extractions steps (as can be seen in Additional files 8, 9). In addition, a large percentage of the GalA is also present in the 1 M KOH, 4 M KOH, and chlorite fraction. This is contrary to the belief that the bulk of pectin is released in wall fractions extracted with ammonium oxalate, CDTA [16], or sodium carbonate. Rather, these data indicate that a significant portion of pectin is tightly held in the wall. This finding is consistent with the recent solid-state nuclear magnetic resonance (SS-NMR) data showing that a substantial portion of pectin (including HG and RG-I) is in close spatial contact with cellulose in Arabidopsis walls [35–37].

Thirdly, the increase and decrease of the Xyl and GalA content in the *PtGAUT12.1*-OE and *PdGAUT12.1*-KD lines, respectively, appeared to be concomitant with trends in the reversed direction of the Man, Gal, and Glc content, i.e., the latter sugars were increased in the KD biomass and decreased in the OE biomass (Table 2, [5], Additional files 8, 9). This was observed both in total AIR and in almost all wall fractions, and was especially obvious in the mass (μg sugar/mg AIR) data. Such trends suggest that the decrease in xylan and HG synthesis by knocking-down *PdGAUT12.1* expression was compensated by increased synthesis of wall polymer(s) that contain Man, Gal, and/or Glc, and vice versa in the *PtGAUT12.1*-OE transgenics. The available sugar linkage data showed mostly similar trends for t-Gal*p*, 4-Man*p*, 4,6-Man*p*, and 4-Glc*p* (Table 3, Additional file 10, [5]), suggesting that the hemicelluloses mannan, including galactomannan and glucomannan, are the likely candidate polymers affected. Indeed, poplar wood contains ~ 5% glucomannan, and a glucomannan synthase gene *CSLA1/GT2A* is known to be highly expressed in poplar during the transition from primary to secondary walls [6, 38, 39]. From the biofuel production perspective, it is conceivable that bioethanol production would benefit from the increased amounts of hexoses Man, Gal, and Glc in the *PdGAUT12.1*-KD biomass compared to controls [5], in addition to the looser walls and increased wall polymer extractability.

Fourthly, it is interesting to note that the Ara and Rha content were negatively affected by both down- and upregulation of *GAUT12.1* expression in the majority of wall fractions of the transgenic biomass (Table 2, Additional files 8, 9, [5]). However, in the total AIR (Table 1, [5]), Ara content was reduced in both KD and OE biomass, while the Rha content of the KD lines was comparable to WT and that of the OE lines was increased compared to WT. The sugar linkage data (Table 3, [5]) showed that t-Ara*f* and 5-Ara*f* sugar linkages were reduced or comparable in the ammonium oxalate and sodium carbonate extracts of both *PdGAUT12.1*-KD and *PtGAUT12.1*-OE biomass, suggesting arabinan as the polymer affected in this case. Such trends suggest indirect effects of the transgenesis on the Ara- and Rha-containing wall polysaccharides.

Fifthly, the glycome profiling data revealed that 4-*O*-methyl-substituted xylan and de-methylesterified HG backbone epitopes increased simultaneously in the oxalate, carbonate, chlorite, and 4 M KOHPC *PtGAUT12.1*-OE wall extracts compared to controls. Although such data are semi-quantitative, it is plausible that the epitopes recognized by these two mAbs groups reside within the same polysaccharide structure, hence facilitating their co-extraction into these wall fractions. The four wall extracts could be investigated further by chromatographic separation coupled with analysis of the fractions by detection of the epitopes using the mAbs in ELISA assays, with the goal of purifying the xylan- and HG-containing polymer(s) for further structural characterization.

**Fig. 11:**
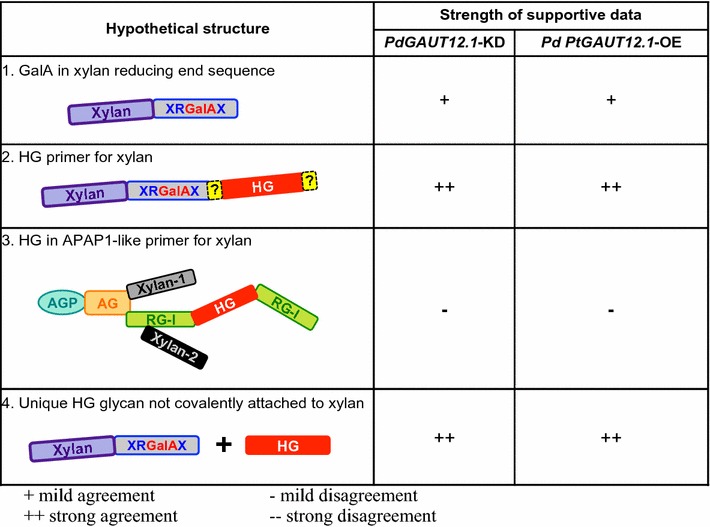
Hypothetical structures that may be synthesized by poplar GAUT12.1. Depiction of four possible structures that could be synthesized by GAUT12 based on available published data on the chemistry and biochemistry of cell walls and cell wall fractions from *GAUT12* mutants and transgenics versus their respective controls. The rectangles depict glycan domains of xylan, the xylan reducing end sequence (XRGalAX), homogalacturonan (HG), arabinogalactan (AG), rhamnogalacturonan I (RG-I), and the two unique xylan regions (Xylan-1, Xylan-2) of the plant proteoglycan Arabinoxylan Pectin Arabinogalactan Protein 1 (APAP1). The oval depicts the arabinogalactan protein (AGP) core of APAP1. The question marks (?) depict hypothetical covalent linkages. Hypothesized function of GAUT12 in structures shown: *structure 1*—insertion of GalA into the xylan reducing end sequence; *structure 2*—synthesis of HG covalently connected directly or indirectly to xylan/xylan reducing end sequence; *structure 3—*synthesis of HG domain in APAP1; *structure 4*—synthesis of HG associated with xylan synthesis but not covalently bound to the xylan polymer. Strength of supportive published data and data from this work: + mild agreement, ++ strong agreement, − mild disagreement; −− strong disagreement

The impacts of modified *GAUT12.1* expression on xylan and HG content led us to hypothesize that GAUT12.1 is a GalAT that catalyzes the incorporation of GalA residues into one or more of four hypothetical structures (Fig. 11): (1) the xylan reducing end sequence, (2) an HG primer for xylan, (3) an HG in an APAP1-like primer for xylan, and (4) a unique HG glycan not covalently attached to xylan. Here we critically evaluate each possible structure based on the combined sugar composition and linkage data from both the *PdGAUT12.1*-KD and *PtGAUT12.1*-OE biomass.

### Hypothetical structure 1

Compromised synthesis of the xylan reducing end sequence would manifest in the KD biomass as a reduction in either total AIR, or in one or more wall fractions, in the amounts of 2-GalA*p*, 3-Rha*p*, and 4-Xyl*p* of the tetrasaccharide sequence, as well as the 4-Xyl*p* of the xylan backbone, and vice versa in the OE biomass. Such effects were observed in the 1 M KOH extracts (Table 3, [5]) that were enriched in hemicellulosic polymers, supporting GAUT12.1 function in the synthesis of the xylan reducing end sequence. However, such a GAUT12.1 function could not explain the substantially altered GalA contents observed in every single wall fraction in both the KD and OE transgenics, thus casting doubt on the role of *GAUT12.1* in the synthesis of the xylan reducing end sequence.

### Hypothetical structure 2

Here we define a xylan structure with an HG primer as a xylan covalently connected to a GAUT12-synthesized HG by yet-to-be-identified glycosyl, base-sensitive, or other linkages. The possibility that GAUT12.1 synthesizes an HG primer for xylan is supported by the concomitant reductions in the amounts of GalA and Xyl in the total AIR and in the 1 M KOH, 4 M KOH, chlorite, 4 M KOHPC, and insoluble wall extracts of the KD biomass (and vice versa for the OE biomass; Tables 1, 2, Additional files 8, 9, [5]), suggesting that such an HG primer for xylan might be recovered in these wall fractions. Furthermore, 4-GalA*p* and 4-Xyl*p* glycosyl linkages were decreased in the 1 M KOH and insoluble wall fractions in the KD biomass and increased in the OE biomass (Table 3, Additional file 10, [5]).

### Hypothetical structure 3

An APAP1-like structure with a GAUT12.1-synthesized HG glycan would be expected to contain 4-GalA*p* for the HG glycan, 2-Rha*p* and/or 2,4-Rha*p* for the RG-I glycan, and 4-Xyl*p* for the xylan glycan. The altered *GAUT12.1* expression would have been expected to affect the contents of these sugars and linkages simultaneously in the wall extracts. We did observe such changes for GalA and Xyl (see description for the “Hypothetical structure 2” above), but not for Rha. The expected trend in Rha content was observed only in the chlorite and insoluble wall fractions, i.e., reduced in the *PdGAUT12.1*-KD and increased in the *PtGAUT12.1*-OE samples, and not in the other *PtGAUT12.1*-OE cell wall fractions. Furthermore, there was not a consistent trend for the 2-Rha*p* and 2,4-Rha*p* linkages in the wall fractions tested (Tables 2, 3, Additional files 8, 9, [5]). Taken together, the results do not support a role for *GAUT12.1* in the synthesis of an APAP1-like structure.

### Hypothetical structure 4

The data showed that GalA content and 4-GalA*p* linkage were affected in all wall extracts for which the sugar composition and linkage data were available (Tables 2, 3, Additional files 8, 9, 10, [5]). These results could be consistent with a function of GAUT12.1 in the synthesis of a unique HG glycan that is not necessarily covalently attached to xylan, but that still affects xylan synthesis.

Based on a consideration of all the available cell wall sugar composition and linkage data from the *PdGAUT12.1*-KD and *PtGAUT12.1*-OE transgenics, the results could support a function for GAUT12 in synthesizing either structures 2 or 4. However, the recovery of increased Xyl in the oxalate and carbonate fractions of the *PdGAUT12.1*-KD lines, while the amount of GalA was decreased in the same fractions [5], leads us to favor hypothetical structure 2 as the polymer synthesized by poplar *GAUT12.1*, i.e., an HG-containing wall polymer that is covalently linked to xylan. We propose that in the *PdGAUT12.1*-KD transgenics, such a structure was not produced at the level required for native wall synthesis, resulting in some xylan not being properly connected in the wall. This scenario is supported by the increased Xyl content and much higher Xyl/GalA ratios in the ammonium oxalate and sodium carbonate wall extracts from *PdGAUT12.1*-KD compared to WT, suggesting that such poorly integrated xylan was released in these wall fractions. We further propose that in the *PtGAUT12.1*-OE plants, overexpression of *PtGAUT12.1* caused increased production of hypothetical structure 2, leading to decreased wall polymer extractability, increased amounts of the insoluble residues remaining after all extractions, increased biomass recalcitrance, and restricted cell and plant growth in the transgenic OE lines. Our data do not show how the HG and xylan are covalently connected in hypothetical structure 2. It is possible that the HG glycan, having been synthesized first, acts as a primer onto which the xylan is synthesized. It is also possible that the HG and xylan glycans are synthesized independently, and subsequently connected together in the secretory pathway. Another alternative is that the HG and xylan are synthesized and secreted into the apoplast independently, and the covalent connection between them occurs in the wall. In the latter two possibilities, the HG glycan of hypothetical structure 2 would serve to anchor the xylan glycan for proper deposition/integration into the wall architecture. Further research is necessary to identify the covalent linkage(s) and the structure/architecture.

Finally, based on the yields of xylan obtained upon extraction of the cell walls with basic solvents, we propose that the covalent connection between the HG and xylan in the GAUT12.1-synthesized structure may be base-sensitive, and thus, is cleaved during the 1 M KOH, 4 M KOH, and 4 M KOHPC extraction steps resulting in high levels of xylan in these fractions. This conclusion is supported by the fact that the greatest increase in GalA content (73–110%) was in the insoluble fractions of the *PtGAUT12.1*-OE biomass compared to controls, while Xyl content in these fractions was only increased by 14–26%. Rather, the bulk of Xyl was recovered in the 1 M KOH wall fractions of the *PtGAUT12.1*-OE biomass compared to controls. Additional supporting evidence for structure 2 was the greater Xyl/GalA ratio in almost all wall fractions of the *PdGAUT12.1*-KD compared to the control, but the similar Xyl/GalA ratios between the *PtGAUT12.1*-OE and control lines (Additional files 8, 9). We propose that in the *PtGAUT12.1*-OE lines, the *Pt*GAUT12.1-synthesized HG was produced in higher amounts, providing more primers/anchors for the xylan to attach to. Therefore, the Xyl/GalA ratios (Additional file 8) would remain largely similar in the different wall extracts of the *PtGAUT12.1*-OE lines and the control. In the *PdGAUT12.1*-KD lines, however, the availability of the *Pd*GAUT12.1-synthesized HG was markedly reduced. We propose that due to reduced amounts of HG primer/anchor, xylan synthesis would still occur but the xylan would not be integrated into the wall properly. As a result, the Xyl/GalA ratios would increase due to an overabundance of xylan compared to the *Pd*GAUT12.1-synthesized HG in almost all wall fractions. Such a result was indeed obtained in the *PdGAUT12.1*-KD lines compared to WT (Additional file 9). Furthermore, as noted above, some xylan would be more easily extracted and be isolated in the oxalate and carbonate fractions. Taken together, the results support the conclusion that poplar GAUT12.1 synthesizes either an HG-containing primer for xylan synthesis or an HG glycan required for proper xylan deposition, anchoring, and/or architecture in the wall, and support the hypothesis that the HG and xylan may be connected to each other by a base-sensitive covalent linkage.

## Conclusions

Carbon-rich hardwood biomass has great potential as a renewable source of material for biofuels and chemicals. A major challenge for use of hardwoods as a feedstock, however, is the recalcitrance of the biomass, which hinders efficient conversion of the polysaccharides to sugars. In this research, we studied the biological function of GAUT12 in plant growth and in modifying biomass quality for saccharification by analyzing the chemical and growth phenotypes of *GAUT12.1* overexpression poplar lines. The resulting *PtGAUT12.1*-OE transgenics had increased recalcitrance and reduced plant growth compared to controls. The stability of the introduced transgene was confirmed by maintenance of the phenotypes in a multi-year field trial. *PtGAUT12.1* overexpression resulted in increased amounts of xylan and HG in the transgenic walls, a concomitant reduction in the extractability of wall material, and increased amounts of cell wall polymers being retained in the final insoluble pellets after sequential extraction of the walls with increasingly harsh solvents. The results indicate that overexpression of *PtGAUT12.1* leads to wall polymers being held more tightly in the walls, and results in reduced saccharification efficiency. Overall, the phenotypes displayed by the *PtGAUT12.1* overexpression lines are opposite to those previously reported for *PdGAUT12.1* knockdown lines [5]. Analysis of the combined comprehensive data from the poplar *GAUT12.1* overexpression and knockdown lines support the hypothesis that poplar GAUT12.1 is involved in the synthesis of a wall structure containing both HG and xylan that may be connected to each other by a base-sensitive covalent linkage.

## Methods

### Generation of the overexpression construct and poplar transgenics lines

A 1602 bp coding sequence of *PtGAUT12.1* (*Potri.001G416800)* was amplified from a *P. trichocarpa* cDNA library via PCR using a primer pair 5′-CACCCCCGGGATGCAGCTTCATATATCGCC-3′ (forward) and 5′-ACGCGTAGTTAAGATGGCCTAATATGACAGC-3′ (reverse), and cloned into pENTR/D-TOPO (Life Technologies). Upon sequence verification, the fragment was transferred into a binary Gateway^®^ destination plasmid using LR Clonase II (Life Technologies) downstream of the *Arabidopsis thaliana Ubiquitin3* promoter. The resulting binary transformation vector, pAGW570 (GenBank accession number MF401557), was transformed into *A. tumefaciens* via electroporation as previously described [5]. The overexpression cassette was subsequently transformed to *P. deltoides* genotype WV94. Presence of the *PtGAUT12.1*-OE construct was verified using PCR from tissue culture shoots.

### Quantitative real time PCR and dot blot analysis

RNA isolation and transcript analyses were performed as previously described [5]. Briefly, the shoot tip from *Populus* (apex), leaf (number 1–3 from top of the plants), internodes (number 1–3 from top of the plants), roots, phloem scraped from the frozen peeled bark, and xylem scraped from the debarked frozen 3-month-old stem were collected and ground to fine powder in liquid nitrogen for transcript analysis. Total RNA was isolated using a CTAB (hexadecyl-trimethylammonium bromide) method as described earlier [5] followed by removal of genomic DNA with DNase (Qiagen, Valencia, CA). First-strand cDNA was synthesized from 1 μg total RNA using the iScript cDNA Synthesis kit (Bio-Rad, Hercules, CA). Quantitative RT-PCR reactions were performed in triplicate using the iQ™ SYBR Green Supermix (Bio-Rad, Hercules, CA) and *18S rRNA* as the reference gene. The relative transcript expression was analyzed as described [5]. Primer sets used are as follows: *GAUT12.1*-F (5′-GGTCGAGCAAAGCCTTGGCTAGATATAGC-3′) and *GAUT12.1*-R (5′-AGATGGCCTAATATGACAGCCCTTTA-3′), and *GAUT12.2*-F (5′-CATTTCAATGGTCGAGCAAAGCCTTGGC-3′) and *GAUT12*-*2*-R (5′-GACAGCCCGTAATGAACTTGTCAGA-3′) [5]. Note that the *GAUT12.1* primer set was expected to recognize both the *P. trichocarpa* transgene and the *P. deltoides* endogenous gene due to the high level of sequence similarity between the two sequences (see “Results” section) and was previously shown to do so [5]. For dot blot analysis, cDNA was synthesized using the total RNA from vascular cambium, xylem, phloem, and leaf tissue. DNA corresponding to nucleotides 1891–2183 of *PtGAUT12.1* transcript (within the 3′-untranslated region) was spotted onto a membrane and probed with the synthesized cDNA samples under stringent conditions as previously described [40].

### Plant growth conditions, growth analysis, and sample isolation

WT and transgenic *P. deltoides* plants were grown in the greenhouse under a 16-h-light/8-h-dark cycle at 25–32 °C, depending on the season, on Fafards 3B soil mix with osmocote, bone meal, gypsum, and dolomite/limestone as previously described [5]. Plants were grown for 9 months. For dry weight measurement, the above ground parts (i.e., entire shoots) of 3-month-old plants were harvested, dried at 70 °C for 5 days, and weighed. Plant water status was measured as relative water content (RWC) from both WT and *PtGAUT12.1*-OE plants as described before [5]. For cell wall, analytical pyrolysis, and saccharification analyses, around ~ 6 cm of the bottom part of the stem was harvested and the bark was peeled. The peeled stem samples were air-dried, the pith removed, the remaining tissues milled to a particle size of 20 mesh (0.85 mm), and the ground samples used for analyses [5].

For the field study, transgenic and control plants were grown in Claxton (GA-30417, USA, 32°9′39″N 81°54′31″W, with a humid subtropical climate) (Additional file 11). Stem radial diameter was measured on 2.8-year-old field-grown transgenic and controls plants. For cell wall analysis, ~ 15 cm of the bottom part of the debarked stem was harvested and air-dried, the pith removed, milled to a particle size of 20 mesh (0.85 mm), and the ground samples used for analysis.

### Cell wall analysis

AIR and fractionated cell walls from ground biomass were prepared as previously described [5]. Glycosyl residue composition analysis of the AIR (~ 2 mg) and cell wall fractions (100–300 µg) from WT and *PtGAUT12.1*-OE samples was performed by combined gas chromatography/mass spectrometry (GC/MS) of the per-*O*-trimethylsilyl (TMS) derivatives of the monosaccharide methyl glycosides produced from the sample by acidic methanolysis as described previously [5, 41, 42]. For glycosyl linkage analysis, samples were permethylated, reduced, re-permethylated, depolymerized, reduced, and acetylated; and the resulting partially methylated alditol acetates (PMAAs) were analyzed by gas chromatography–mass spectrometry (GC–MS) as described [5, 43].

### Lignin analysis and saccharification assay

The National Renewable Energy Laboratory (NREL) high-throughput pyrolysis molecular beam mass spectrometry (MBMS) method was used to quantify lignin content and S/G lignin monomer ratio from WT and *PtGAUT12.1*-OE biomass as previously described [5, 44, 45]. For the saccharification assay, the NREL high-throughput thermochemical pretreatment and enzymatic hydrolysis sugar release assay was carried out as previously described [5, 46, 47].

### Glycome profiling

Sequential cell walls extracted fractions from *Populus* WT and *PtGAUT12.1*-OE lines were subjected to glycome profiling analysis as described [5, 25]. The presence of epitopes recognized by Enzyme-Linked Immunosorbent Assay (ELISA)-based monoclonal antibodies (mAb) screenings were represented as heat maps.

### Microscopy

Tissue fixation and embedding, maceration of xylem, and microscopy analyses were performed as described [5].

### Statistical analysis

Statistical analysis was performed using Statistica 5.0. The significance of differences between control and transgenic samples was analyzed using a one-way ANOVA followed by Tukey’s multiple comparison test.

## Additional files

**Additional file 1.** **a** Glucose, **b** xylose, and **c** total sugar release from *P. deltoides* wild-type (WT), vector control and *PtGAUT12*-OE lines. *n* = 25 for WT, *n* = 10–15 for each vector control and *PtGAUT12*-OE lines. Significance *P* values are expressed as *P < 0.05, **P < 0.001 by one-way analysis of variance (ANOVA) followed by Tukey’s multiple comparison test using Statistica 5.0.

**Additional file 2.** Total lignin content and S/G ratio of *P. deltoides* wild-type (WT), vector control and *PtGAUT12.1*-OE lines. Values are mean ± SE, *n* = 25 for WT, *n* = 10–15 for vector control (V. Control-1-8) and *PtGAUT12.1*-OE lines (AB29.1–AB29.13). In bold and denoted with a star are transgenic values that are significantly different from WT and vector control lines at *P* < 0.05, as determined by one-way analysis of variance (ANOVA) followed by Tukey’s multiple comparison test using Statistica 5.0.

**Additional file 3.** Plant height and diameter of three-month-old *P. deltoides* wild-type (WT), vector control and *PtGAUT12.1*-OE lines. Values are mean ± SE, *n* = 25 for WT, *n* = 10–15 for vector control (V. Control-1-8) and *PtGAUT12.1*-OE lines (AB29.1–AB29.13). Transgenic values that are significantly different from WT and vector control lines are in bold and denoted with one star (*P* < 0.05) or two stars (*P* < 0.001), as determined by one-way analysis of variance (ANOVA) followed by Tukey’s multiple comparison test using Statistica 5.0.

**Additional file 4.** Correlation between the total *GAUT12.1* transcript expression and plant growth in *P. deltoides PtGAUT12.1*-OE transgenic and control lines. **a** Height and **b** radial diameter of 3-month-old greenhouse-grown poplar plants are plotted against the transcript expression level. Blue diamonds—WT; black diamonds—vector controls; red diamonds—*PtGAUT12.1*-OE lines (please note that on the graphs the WT blue diamonds may be obscured by the vector control black diamonds). *n* = 25 for WT, *n* = 10–15 for vector control and *PtGAUT12.1*-OE lines.

**Additional file 5.** Leaf phenotypes of 3-month-old, greenhouse-grown *P. deltoides PtGAUT12.1*-OE lines compared to controls. **a** Comparison of leaves (the sixth leaf from apex) from *P. deltoides* WT, vector control, and *PtGAUT12.1*-OE plants. **b** Length and **c** width of leaves of different developmental stages from 3-month-old plants. Every third leaf from the apex of ten plants was measured. **d** Developing (10th leaf from apex) and **e** fully expanded (20th leaf from apex) leaf areas measured from five plants. **f** The relative water content (RWC) of WT and *PtGAUT12.1*-OE lines. Error bars represent SE. *P < 0.05, **P < 0.001. **g**, **h** Correlation between RWC at 72 h and fully expanded leaf area of WT and *P. deltoides PdGAUT12.1*-KD (**g**) and *PtGAUT12.1*-OE (**h**) lines.

**Additional file 6.** Glycosyl residue composition of (**a**) alcohol insoluble residue (AIR) and (**b**–**h**) wall fractions from stems of field-grown *P. deltoides* control and *PtGAUT12.1*-OE transgenic plants. Wall fractions were prepared by sequential extraction of AIR using increasingly harsh reagents: (**b**) 50 mM ammonium oxalate, (**c**) 50 mM Na_2_CO_3_, (**d**) 1 M KOH, (**e**) 4 M KOH, (**f**) 100 mM sodium chlorite (chlorite) and (**g**) 4 M KOH post-chlorite (4 M KOH PC). (**h**) The insoluble pellet remaining after all the extractions. Glycosyl residue composition was determined by GC–MS of trimetylsilyl (TMS) derivatives. Data are mean ± SE of three biological and two technical replicates, *n* = 5. *P < 0.05, **P < 0.001.

**Additional file 7.** Mass of AIR, total material recovered in sequential AIR extracts and in the insoluble pellet from *P. deltoides PtGAUT12.1*-OE lines compared to controls. **a** Mass of AIR extracted per gram of ground dry stem tissues of *PtGAUT12.1*-OE and control lines. **b**–**g** Amount of material recovered in wall fractions extracted by **b** 50 mM ammonium oxalate, **c** 50 mM sodium carbonate, **d** 1 M KOH, **e** 4 M KOH, **f** 100 mM sodium chlorite, **g** 4 M KOH post chlorite (PC). **h** Total amount of material recovered in all wall fractions combined. **i** Amount of material remaining in the insoluble pellet, after all the extractions. Data in panels **b** through **i** are average mg extract per gram AIR ± SE, *n* = 4. Significance *P* values are expressed as *P < 0.05, **P < 0.001.

**Additional file 8.** Glycosyl residue composition and total carbohydrate of cell wall extracts from stem tissues of 9-month-old, greenhouse-grown *P. deltoides* WT and *PtGAUT12.1*-OE lines (AB29.2, AB29.7, and AB29.12). Data are average μg amount of each designated monosaccharide per mg alcohol insoluble residue (AIR), measured by GC–MS of tetramethylsilane (TMS) derivatives. Wall extracts are from sequential extractions of AIR using 50 mM ammonium oxalate, 50 mM sodium carbonate, 1 M KOH, 4 M KOH, 100 mM sodium chlorite (chlorite), 4 M KOH post-chlorite (PC), and the final residual insoluble pellet. Values in bold are those of the transgenic lines that are significantly different from WT values at *P < 0.05 and **P < 0.001 significant levels (one-way ANOVA followed by Tukey’s multiple comparison test).

**Additional file 9.** Glycosyl residue composition and total carbohydrate of cell wall extracts from stem tissues of 9-month-old, greenhouse-grown *P. deltoides* WT and *PdGAUT12.1*-KD lines (AB30.1, AB30.3, AB30.8 and AB30.11). Data are average μg amount of each designated monosaccharide per mg alcohol insoluble residue (AIR), measured by GC–MS of tetramethylsilane (TMS) derivatives. Wall extracts are from sequential extractions of AIR using 50 mM ammonium oxalate, 50 mM sodium carbonate, 1 M KOH, 4 M KOH, 100 mM sodium chlorite (chlorite), 4 M KOH post-chlorite (PC), and the final residual insoluble pellet. The data, except for those of the insoluble wall fractions, were previously reported in mol% and mass (μg sugar/mg extract) [5]. Values in bold are those of the transgenic lines that are significantly different from WT values at *P < 0.05 and **P < 0.001 significant levels (one-way ANOVA followed by Tukey’s multiple comparison test).

**Additional file 10.** Glycosyl linkage analysis of the residual insoluble cell wall that remained after sequential extractions of AIR from *P. deltoides* WT, *PdGAUT12.1*-KD, and *PtGAUT12.1*-OE lines. Data are in mol percentages, provided by CCRC Analytical Services.

**Additional file 11.** Field design of the multi-year field trial of *P. deltoides PtGAUT12.1*-OE and control lines. The field was divided into three replicate plots, each contained completely randomized genotype subplots. Each subplot contained two vegetatively propagated clonal trees, planted 3 m × 3 m apart from each other and from other trees in the neighboring subplots. Border trees were also planted surrounding the whole plot to control for shading effects. Grey boxes represent subplots containing transgenics lines with genotypes other than the *PtGAUT12.1*-OE, vector control, and WT lines.

## Abbreviations

AIR: alcohol insoluble residue
GalA: galacturonic acid
GAUT1: GAlactUronosylTransferase1
GAUT12: GAlactUronosylTransferase12
GlcA: glucuronic acid
GT8: glycosyltransferase family 8
GX: glucuronoxylan
HG: homogalacturonan
MBMS: molecular beam mass spectrometry
MeGlcA: methyl glucuronic acid
RG: rhamnogalacturonan
TMS: trimethylsilyl
WT: wild type
