# Supplementary material for: Working towards recalcitrance mechanisms: increased xylan and homogalacturonan production by overexpression of GAlactUronosylTransferase12 (GAUT12) causes increased recalcitrance and decreased growth in Populus
Source: Biotechnol Biofuels. 2018 Jan 17;11:9. doi: 10.1186/s13068-017-1002-y (PMC5771077; doi:10.1186/s13068-017-1002-y)
Supplement: Supplementary file 1 — Additional file 1. a Glucose, b xylose, and c total sugar release from P. deltoides wild-type (WT), vector control and PtGAUT12-OE lines. n = 25 for WT, n = 10–15 for each vector control and PtGAUT12-OE lines. Significance P values are expressed as *P < 0.05, **P < 0.001 by one-way analysis of variance (ANOVA) followed by Tukey’s multiple comparison test using Statistica 5.0. [file 13068_2017_1002_MOESM1_ESM.docx]

**Additional file 1.** (**a**) Glucose, (**b**) xylose, and (**c**) total sugar release from *P. deltoides* wild-type (WT), vector control and *PtGAUT12.1*-OE lines. *n* = 25 for WT, *n* = 10–15 for each vector control and *PtGAUT12.1*-OE lines. Significance *P* values are expressed as **P* < 0.05, ***P* < 0.001 by one-way analysis of variance (ANOVA) followed by Tukey’s multiple comparison test using Statistica 5.0.
