# Supplementary material for: Working towards recalcitrance mechanisms: increased xylan and homogalacturonan production by overexpression of GAlactUronosylTransferase12 (GAUT12) causes increased recalcitrance and decreased growth in Populus
Source: Biotechnol Biofuels. 2018 Jan 17;11:9. doi: 10.1186/s13068-017-1002-y (PMC5771077; doi:10.1186/s13068-017-1002-y)
Supplement: Supplementary file 2 — Additional file 2. Total lignin content and S/G ratio of P. deltoides wild-type (WT), vector control and PtGAUT12.1-OE lines. Values are mean ± SE, n = 25 for WT, n = 10–15 for vector control (V. Control-1-8) and PtGAUT12.1-OE lines (AB29.1–AB29.13). In bold and denoted with a star are transgenic values that are significantly different from WT and vector control lines at P < 0.05, as determined by one-way analysis of variance (ANOVA) followed by Tukey’s multiple comparison test using Statistica 5.0. [file 13068_2017_1002_MOESM2_ESM.docx]

**Additional file 2 -** Total lignin content and S/G ratio of *P. deltoides* wild-type (WT), vector control and *PtGAUT12.1-*OE lines. Values are means ± SE, n = 25 for WT, n = 10-15 for vector control (V Control-1-8) and *PtGAUT12.1-*OE lines (AB29.1-AB29.13). In bold and denoted with a star are transgenic values that are significantly different from WT and vector control lines at *P* < 0.05, as determined by one-way analysis of variance (ANOVA) followed by Tukey’s multiple comparison test using Statistica 5.0.

| **Genotype** | **Lignin content (%)** | **S/G ratio** |
| --- | --- | --- |
| WT | 25.66 ± 0.17 | 1.81 ± 0.03 |
| V Control-1 | 25.63 ± 0.22 | 1.84 ± 0.01 |
| V Control-2 | 26.61 ± 0.19 | 1.82 ± 0.04 |
| V Control-3 | 25.71 ± 0.28 | 1.78 ± 0.03 |
| V Control-4 | 26.72 ± 0.36 | 1.83 ± 0.03 |
| V Control-5 | 26.02 ± 0.31 | 1.82 ± 0.04 |
| V Control-6 | 25.66 ± 0.56 | 1.81 ± 0.03 |
| V Control-7 | 25.82 ± 0.34 | 1.77 ± 0.02 |
| V Control-8 | 24.71 ± 0.44 | 1.78 ± 0.03 |
| AB29.1 | 25.48 ± 0.18 | 1.82 ± 0.02 |
| AB29.2 | 25.59 ± 0.38 | **1.67 ± 0.02*** |
| AB29.3 | 25.95 ± 0.44 | 1.80 ± 0.03 |
| AB29.4 | 25.61 ± 0.18 | 1.82 ± 0.04 |
| AB29.5 | 25.62 ± 0.34 | 1.82 ± 0.03 |
| AB29.6 | 26.01 ± 0.23 | 1.81 ± 0.03 |
| AB29.7 | 25.23 ± 0.41 | **1.61 ± 0.01*** |
| AB29.8 | 25.84 ± 0.26 | 1.79 ± 0.04 |
| AB29.9 | 26.01 ± 0.22 | **1.63 ± 0.01*** |
| AB29.10 | 26.27 ± 0.43 | **1.67 ± 0.02*** |
| AB29.11 | 25.82 ± 0.32 | 1.82 ± 0.01 |
| AB29.12 | 25.31 ± 0.35 | **1.67 ± 0.02*** |
| AB29.13 | 25.86 ± 0.28 | 1.78 ± 0.04 |
