# Supplementary material for: Working towards recalcitrance mechanisms: increased xylan and homogalacturonan production by overexpression of GAlactUronosylTransferase12 (GAUT12) causes increased recalcitrance and decreased growth in Populus
Source: Biotechnol Biofuels. 2018 Jan 17;11:9. doi: 10.1186/s13068-017-1002-y (PMC5771077; doi:10.1186/s13068-017-1002-y)
Supplement: Supplementary file 3 — Additional file 3. Plant height and diameter of three-month-old P. deltoides wild-type (WT), vector control and PtGAUT12.1-OE lines. Values are mean ± SE, n = 25 for WT, n = 10–15 for vector control (V. Control-1-8) and PtGAUT12.1-OE lines (AB29.1–AB29.13). Transgenic values that are significantly different from WT and vector control lines are in bold and denoted with one star (P < 0.05) or two stars (P < 0.001), as determined by one-way analysis of variance (ANOVA) followed by Tukey’s multiple comparison test using Statistica 5.0. [file 13068_2017_1002_MOESM3_ESM.docx]

**Additional file 3 -** Plant height and diameter of three-month-old P. deltoides wild-type (WT), vector control and PtGAUT12.1-OE lines. Values are means ± SE, n = 25 for WT, n = 10-15 for vector control (V Control-1-8) and *PtGAUT12.1-*OE lines (AB29.1-AB29.13). Transgenic values that are significantly different from WT and vector control lines are in bold and denoted with one star (*P* < 0.05) or two stars (*P* < 0.001), as determined by one-way analysis of variance (ANOVA) followed by Tukey’s multiple comparison test using Statistica 5.0.

| **Genotype** | **Plant height (inches)** | **Plant diameter (mm)** |
| --- | --- | --- |
| WT | 47.56 ± 2.7 | 6.2 ± 0.12 |
| V Control-1 | 47.23 ± 1.9 | 5.9 ± 0.22 |
| V Control-2 | 48.51 ± 2.3 | 6.1 ± 0.21 |
| V Control-3 | 45.98 ± 2.5 | 6.2 ± 0.18 |
| V Control-4 | 48.21 ± 2.7 | 5.9 ± 0.23 |
| V Control-5 | 47.56 ± 1.1 | 6.3 ± 0.25 |
| V Control-6 | 46.88 ± 2.0 | 6.2 ± 0.31 |
| V Control-7 | 47.47 ± 1.3 | 6.1 ± 0.32 |
| V Control-8 | 46.83 ± 1.4 | 6.2 ± 0.24 |
| AB29.1 | 49.13 ± 1.3 | 6.4 ± 0.17 |
| AB29.2 | **24.41 ± 1.0**** | **4.2 ± 0.16**** |
| AB29.3 | **41.32 ± 1.2*** | **5.6 ± 0.21*** |
| AB29.4 | **40.88 ± 1.1*** | **5.7 ± 0.11*** |
| AB29.5 | **44.72 ± 0.8*** | **5.7 ± 0.31*** |
| AB29.6 | **41.02 ± 1.3*** | **5.6 ± 0.12*** |
| AB29.7 | **24.18 ± 1.2**** | **4.1 ± 0.11**** |
| AB29.8 | 49.07 ± 1.4 | 6.1 ± 0.17 |
| AB29.9 | **33.50 ± 0.9**** | **4.9 ± 0.10**** |
| AB29.10 | 47.00 ± 1.0 | 6.1 ± 0.18 |
| AB29.11 | 47.89 ± 1.1 | 6.3 ± 0.21 |
| AB29.12 | **21.95 ± 0.8**** | **3.7 ± 0.17**** |
| AB29.13 | **44.11 ± 1.1*** | **5.7 ± 0.24*** |
