# Supplementary material for: Working towards recalcitrance mechanisms: increased xylan and homogalacturonan production by overexpression of GAlactUronosylTransferase12 (GAUT12) causes increased recalcitrance and decreased growth in Populus
Source: Biotechnol Biofuels. 2018 Jan 17;11:9. doi: 10.1186/s13068-017-1002-y (PMC5771077; doi:10.1186/s13068-017-1002-y)
Supplement: Supplementary file 4 — Additional file 4. Correlation between the total GAUT12.1 transcript expression and plant growth in P. deltoides PtGAUT12.1-OE transgenic and control lines. a Height and b radial diameter of 3-month-old greenhouse-grown poplar plants are plotted against the transcript expression level. Blue diamonds—WT; black diamonds—vector controls; red diamonds—PtGAUT12.1-OE lines (please note that on the graphs the WT blue diamonds may be obscured by the vector control black diamonds). n = 25 for WT, n = 10–15 for vector control and PtGAUT12.1-OE lines. [file 13068_2017_1002_MOESM4_ESM.docx]

**Additional file 4.** Correlation between the total *GAUT12.1* transcript expression and plant growth in *P. deltoides PtGAUT12.1*-OE transgenic and control lines. (**a**) Height and (**b**) radial diameter of 3-month-old greenhouse-grown poplar plants are plotted against the transcript expression level. Blue diamonds—WT; black diamonds—vector controls; red diamonds—*PtGAUT12.1*-OE lines (please note that on the graphs the WT blue diamonds may be obscured by the vector control black diamonds). *n* = 25 for WT, *n* = 10–15 for vector control and *PtGAUT12.1*-OE lines.
