# Supplementary material for: Working towards recalcitrance mechanisms: increased xylan and homogalacturonan production by overexpression of GAlactUronosylTransferase12 (GAUT12) causes increased recalcitrance and decreased growth in Populus
Source: Biotechnol Biofuels. 2018 Jan 17;11:9. doi: 10.1186/s13068-017-1002-y (PMC5771077; doi:10.1186/s13068-017-1002-y)
Supplement: Supplementary file 5 — Additional file 5. Leaf phenotypes of 3-month-old, greenhouse-grown P. deltoides PtGAUT12.1-OE lines compared to controls. a Comparison of leaves (the sixth leaf from apex) from P. deltoides WT, vector control, and PtGAUT12.1-OE plants. b Length and c width of leaves of different developmental stages from 3-month-old plants. Every third leaf from the apex of ten plants was measured. d Developing (10th leaf from apex) and e fully expanded (20th leaf from apex) leaf areas measured from five plants. f The relative water content (RWC) of WT and PtGAUT12.1-OE lines. Error bars represent SE. *P < 0.05, **P < 0.001. g, h Correlation between RWC at 72 h and fully expanded leaf area of WT and P. deltoides PdGAUT12.1-KD (g) and PtGAUT12.1-OE (h) lines. [file 13068_2017_1002_MOESM5_ESM.docx]

**Additional file 5.** Leaf phenotypes of 3-month-old, greenhouse-grown *P. deltoides PtGAUT12.1*-OE lines compared to controls. (**a**) Comparison of leaves (the sixth leaf from apex) from *P. deltoides* WT, vector control, and *PtGAUT12.1*-OE plants. (**b**) Length and (**c**) width of leaves of different developmental stages from 3-month-old plants. Every third leaf from the apex of ten plants was measured. (**d**) Developing (10^th^ leaf from apex) and (**e**) fully expanded (20^th^ leaf from apex) leaf areas measured from five plants. (**f**) The relative water content (RWC) of WT and *PtGAUT12.1*-OE lines. Error bars represent SE. **P* < 0.05, ***P* < 0.001. (**g-h**) Correlation between RWC at 72 h and fully expanded leaf area of WT and *P. deltoides* *PdGAUT12.1*-KD (**g**) and *PtGAUT12.1*-OE (**h**) lines.
