# Supplementary material for: Working towards recalcitrance mechanisms: increased xylan and homogalacturonan production by overexpression of GAlactUronosylTransferase12 (GAUT12) causes increased recalcitrance and decreased growth in Populus
Source: Biotechnol Biofuels. 2018 Jan 17;11:9. doi: 10.1186/s13068-017-1002-y (PMC5771077; doi:10.1186/s13068-017-1002-y)
Supplement: Supplementary file 6 — Additional file 6. Glycosyl residue composition of (a) alcohol insoluble residue (AIR) and (b–h) wall fractions from stems of field-grown P. deltoides control and PtGAUT12.1-OE transgenic plants. Wall fractions were prepared by sequential extraction of AIR using increasingly harsh reagents: (b) 50 mM ammonium oxalate, (c) 50 mM Na2CO3, (d) 1 M KOH, (e) 4 M KOH, (f) 100 mM sodium chlorite (chlorite) and (g) 4 M KOH post-chlorite (4 M KOH PC). (h) The insoluble pellet remaining after all the extractions. Glycosyl residue composition was determined by GC–MS of trimetylsilyl (TMS) derivatives. Data are mean ± SE of three biological and two technical replicates, n = 5. *P < 0.05, **P < 0.001. [file 13068_2017_1002_MOESM6_ESM.docx]

**Additional file 6.** Glycosyl residue composition of (**a**) alcohol insoluble residue (AIR) and (**b-h**) wall fractions from stems of field-grown *P. deltoides* control and *PtGAUT12.1*-OE transgenic plants. Wall fractions were prepared by sequential extraction of AIR using increasingly harsh reagents: (**b**) 50 mM ammonium oxalate, (**c**) 50 mM Na_2_CO_3_, (**d**) 1 M KOH, (**e**) 4 M KOH, (**f**) 100 mM sodium chlorite (chlorite) and (**g**) 4 M KOH post-chlorite (4 M KOH PC). (**h**) The insoluble pellet remaining after all the extractions. Glycosyl residue composition was determined by GC–MS of trimetylsilyl (TMS) derivatives. Data are mean ± SE of three biological and two technical replicates, *n* = 5. **P* < 0.05, ***P* < 0.001.
