# Supplementary material for: Working towards recalcitrance mechanisms: increased xylan and homogalacturonan production by overexpression of GAlactUronosylTransferase12 (GAUT12) causes increased recalcitrance and decreased growth in Populus
Source: Biotechnol Biofuels. 2018 Jan 17;11:9. doi: 10.1186/s13068-017-1002-y (PMC5771077; doi:10.1186/s13068-017-1002-y)
Supplement: Supplementary file 7 — Additional file 7. Mass of AIR, total material recovered in sequential AIR extracts and in the insoluble pellet from P. deltoides PtGAUT12.1-OE lines compared to controls. a Mass of AIR extracted per gram of ground dry stem tissues of PtGAUT12.1-OE and control lines. b–g Amount of material recovered in wall fractions extracted by b 50 mM ammonium oxalate, c 50 mM sodium carbonate, d 1 M KOH, e 4 M KOH, f 100 mM sodium chlorite, g 4 M KOH post chlorite (PC). h Total amount of material recovered in all wall fractions combined. i Amount of material remaining in the insoluble pellet, after all the extractions. Data in panels b through i are average mg extract per gram AIR ± SE, n = 4. Significance P values are expressed as *P < 0.05, **P < 0.001. [file 13068_2017_1002_MOESM7_ESM.docx]

**Additional file 7.** Mass of AIR, total material recovered in sequential AIR extracts and in the insoluble pellet from *P. deltoides PtGAUT12.1*-OE lines compared to controls. (**a**) Mass of AIR extracted per gram from ground dry stem tissues of *PtGAUT12.1*-OE and control lines. (**b–g**) Amount of material recovered in wall fractions extracted by (**b**) 50 mM ammonium oxalate, (**c**) 50 mM sodium carbonate, (**d**) 1 M KOH, (**e**) 4 M KOH, (**f**) 100 mM sodium chlorite, (**g**) 4 M KOH post chlorite (PC). (**h**) Total amount of material recovered in all wall fractions combined. (**i**) Amount of material remaining in the insoluble pellet, after all the extractions. Data in panels **b** through **i** are average mg extract per gram AIR ± SE, *n* = 4. Significance *P* values are expressed as **P* < 0.05, ***P* < 0.001.
