# Supplementary material for: Working towards recalcitrance mechanisms: increased xylan and homogalacturonan production by overexpression of GAlactUronosylTransferase12 (GAUT12) causes increased recalcitrance and decreased growth in Populus
Source: Biotechnol Biofuels. 2018 Jan 17;11:9. doi: 10.1186/s13068-017-1002-y (PMC5771077; doi:10.1186/s13068-017-1002-y)
Supplement: Supplementary file 10 — Additional file 10. Glycosyl linkage analysis of the residual insoluble cell wall that remained after sequential extractions of AIR from P. deltoides WT, PdGAUT12.1-KD, and PtGAUT12.1-OE lines. Data are in mol percentages, provided by CCRC Analytical Services. [file 13068_2017_1002_MOESM10_ESM.docx]

**Additional file 10 -** Glycosyl linkage analysis of the residual insoluble cell wall that remained after sequential extractions of AIR from *P. deltoides* WT, *PdGAUT12.1*-KD, and *PtGAUT12.1*-OE lines. Data are in mol percentages, provided by CCRC Analytical Services.

|  | **Insoluble** | | | | |
| --- | --- | --- | --- | --- | --- |
|  | **WT** | ***PdGAUT12.1*-KD** | | ***Pd PtGAUT12.1*-OE** | |
|  |  | **AB30.1** | **AB30.8** | **AB29.2** | **AB29.12** |
| 4-Ara*p* or 5-Ara*f* | 0.3 | 0.4 | 0.3 | 0.3 | 0.3 |
| 2-Rha*p* | 0.2 | 0.2 | 0.2 | 0.2 | 0.3 |
| 2,4-Rha*p* | 0.4 | 0.2 | 0.2 | 0.6 | 0.5 |
| 4-Xyl*p* | 0.6 | 0.4 | 0.3 | 0.8 | 0.8 |
| t-GlcA*p* | 0.3 | 0.2 | 0.1 | 0.4 | 0.4 |
| t-GalA*p* | 0.4 | 0.3 | 0.2 | 0.6 | 0.7 |
| 4-GalA*p* | 1.0 | 0.4 | 0.2 | 1.4 | 1.5 |
| t-Man*p* | 1.1 | 1.0 | 1.0 | 1.0 | 1.0 |
| 4-Man*p* | 42.1 | 39.0 | 39.2 | 44.8 | 47.2 |
| 4,6-Man*p* | 0.4 | 0.4 | 0.3 | 0.2 | 0.2 |
| t-Gal*p* | 1.6 | 1.7 | 2.1 | 0.3 | 0.4 |
| 4-Gal*p* | 1.1 | 1.2 | 1.8 | 0.6 | 0.5 |
| 2,4-Gal*p* | 0.2 | 0.3 | 0.8 | 0.1 | 0.1 |
| t-Glc*p* | 2.9 | 2.6 | 2.5 | 3.1 | 3.3 |
| 4-Glc*p* | 47.1 | 51.5 | 50.6 | 45.5 | 42.7 |
| 3,4-Glc*p* | 0.3 | 0.2 | 0.2 | 0.1 | 0.1 |
