# Supplementary material for: Working towards recalcitrance mechanisms: increased xylan and homogalacturonan production by overexpression of GAlactUronosylTransferase12 (GAUT12) causes increased recalcitrance and decreased growth in Populus
Source: Biotechnol Biofuels. 2018 Jan 17;11:9. doi: 10.1186/s13068-017-1002-y (PMC5771077; doi:10.1186/s13068-017-1002-y)
Supplement: Supplementary file 11 — Additional file 11. Field design of the multi-year field trial of P. deltoides PtGAUT12.1-OE and control lines. The field was divided into three replicate plots, each contained completely randomized genotype subplots. Each subplot contained two vegetatively propagated clonal trees, planted 3 m × 3 m apart from each other and from other trees in the neighboring subplots. Border trees were also planted surrounding the whole plot to control for shading effects. Grey boxes represent subplots containing transgenics lines with genotypes other than the PtGAUT12.1-OE, vector control, and WT lines. [file 13068_2017_1002_MOESM11_ESM.docx]

**Additional file 11 –** Field design of the multi-year field trial of *P. deltoides PtGAUT12.1*-OE and control lines. The field was divided into three replicate plots, each contained completely randomized genotype subplots. Each subplot contained two vegetatively propagated clonal trees, planted 3 m x 3 m apart to each other and to other trees in the neighboring subplots. Border trees were also planted surrounding the whole plot to control for shading effects. Grey boxes represent subplots containing transgenics lines with genotypes other than the *PtGAUT12.1*-OE, vector control, and WT lines.
